# Monoclonal antibody Y01 prevents tauopathy progression induced by lysine 280–acetylated tau in cell and mouse models

**DOI:** 10.1172/JCI156537

**Published:** 2023-04-17

**Authors:** Ha-Lim Song, Na-Young Kim, Jaewan Park, Meong Il Kim, Yu-Na Jeon, Se-Jong Lee, Kwangmin Cho, Young-Lim Shim, Kyoung-Hye Lee, Yeon-Seon Mun, Jung-A Song, Min-Seok Kim, Chan-Gi Pack, Minkyo Jung, Hyemin Jang, Duk L. Na, Minsun Hong, Dong-Hou Kim, Seung-Yong Yoon

**Affiliations:** 1 ADEL Institute of Science & Technology (AIST), ADEL Inc., Seoul, South Korea.; 2 Division of Biological Science and Technology, Yonsei University, Wonju, South Korea.; 3 Department of Brain Science and; 4 Department of Convergence Medicine, Asan Medical Center, University of Ulsan College of Medicine, Seoul, South Korea.; 5 Neural Circuits Research Group, Korea Brain Research Institute, Daegu, South Korea.; 6 Samsung Alzheimer’s Convergence Research Center, Department of Neurology, Neuroscience Center, Samsung Medical Center, Samsung Advanced Institute for Health Sciences and Technology, Sungkyunkwan University School of Medicine, Seoul, South Korea.; 7 Stem Cell Immunomodulation Research Center, University of Ulsan College of Medicine, Seoul, South Korea.

**Keywords:** Neuroscience, Therapeutics, Alzheimer disease, Immunotherapy

## Abstract

The spatiotemporal pattern of the spread of pathologically modified tau through brain regions in Alzheimer’s disease (AD) can be explained by prion-like cell-to-cell seeding and propagation of misfolded tau aggregates. Hence, to develop targeted therapeutic antibodies, it is important to identify the seeding- and propagation-competent tau species. The hexapeptide ^275^VQIINK^280^ of tau is a critical region for tau aggregation, and K280 is acetylated in various tauopathies, including AD. However, the mechanism that links tau acetylated on lysine 280 (tau-acK280) to subsequent progression to neurodegenerative disease remains unclear. Here, we demonstrate that tau-acK280 is critical for tau propagation processes including secretion, aggregation, and seeding. We developed an antibody, Y01, that specifically targets tau-acK280 and solved the crystal structure of Y01 in complex with an acK280 peptide. The structure confirmed that Y01 directly recognizes acK280 and the surrounding residues. Strikingly, upon interaction with acetylated tau aggregates, Y01 prevented tauopathy progression and increased neuronal viability in neuron cultures and in tau-Tg mice through antibody-mediated neutralization and phagocytosis, respectively. Based on our observations that tau-acK280 is a core species involved in seeding and propagation activities, the Y01 antibody that specifically recognizes acK280 represents a promising therapeutic candidate for AD and other neurodegenerative diseases associated with tauopathy.

## Graphical abstract

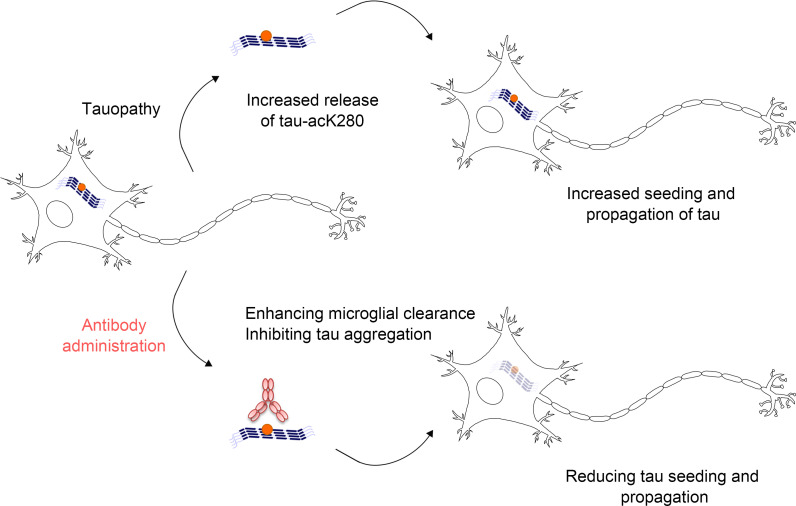

## Introduction

Tau, a naturally soluble protein in cells of the central nervous system, promotes microtubule assembly in healthy individuals (1, 2). Hereditary mutations or posttranslational modifications (PTMs) cause soluble tau molecules to transform into filamentous aggregates. The resultant tauopathies, which are pathological conditions responsible for many neurodegenerative diseases (3), are classified into 2 types based on discrete target locations and clinical signs. Primary tauopathies, characterized by neuronal and glial tau inclusions, are associated with neurodegenerative diseases such as progressive supranuclear palsy, Pick’s disease, and corticobasal degeneration (CBD) (4). By contrast, secondary tauopathy is associated with extracellular β-amyloid plaques of Alzheimer’s disease (AD) (5).

In tauopathy-linked diseases, tau exists as multiple heterologous species (proteoforms) comprising polypeptides and chemical compositions resulting from different isoforms and various PTMs (6–9). In many neurodegenerative diseases, tau proteoforms cause irreversible conformational shifts from soluble monomers to laterally stackable aggregates (10). Six tau isoforms have been described, with 0, 1, or 2 N-terminal acidic inserts (0N, 1N, or 2N, respectively) and 3 or 4 microtubule-binding repeats (MTBRs) (3R or 4R) (2). In addition to the multiple isoforms derived from genetic variation, tau is subjected to various PTMs, including phosphorylation, ubiquitination, acetylation, and methylation (11), which makes it more challenging to select the most effective epitope for tau in contrast to short β-amyloid (Aβ). Following pathogenic PTM, tau forms neurofibrils in brain regions including the transentorhinal cortex, and then spreads into connected recipient neurons in other brain areas, leading to progressive AD (12–14). This phenomenon indicates that pathogenic tau species seed soluble tau strains into abnormal tau aggregates in a prion-like manner. For example, tau filaments can be visualized in the brains of patients with CBD or AD (15). Acetylation, recently identified as a key pathogenic PTM, decreases tau binding to microtubules, increases tau aggregation, decreases tau degradation, and leads to synaptic dysfunction and loss (8, 16, 17).

Various tau species and modifications have been reported to be related to AD, but it is unclear which forms are true therapeutic targets rather than merely being pathologically correlated findings. For early diagnosis of AD and other neurodegenerative diseases, as well as the development of target-specific therapies for these disorders, it is necessary to understand at the molecular and cellular levels how tau initiates the formation of detrimental tauopathies. We addressed this issue, developed an antibody, and evaluated its efficacy.

## Results

### Tau secretion and propagation by acetylation.

**Figure 1:**
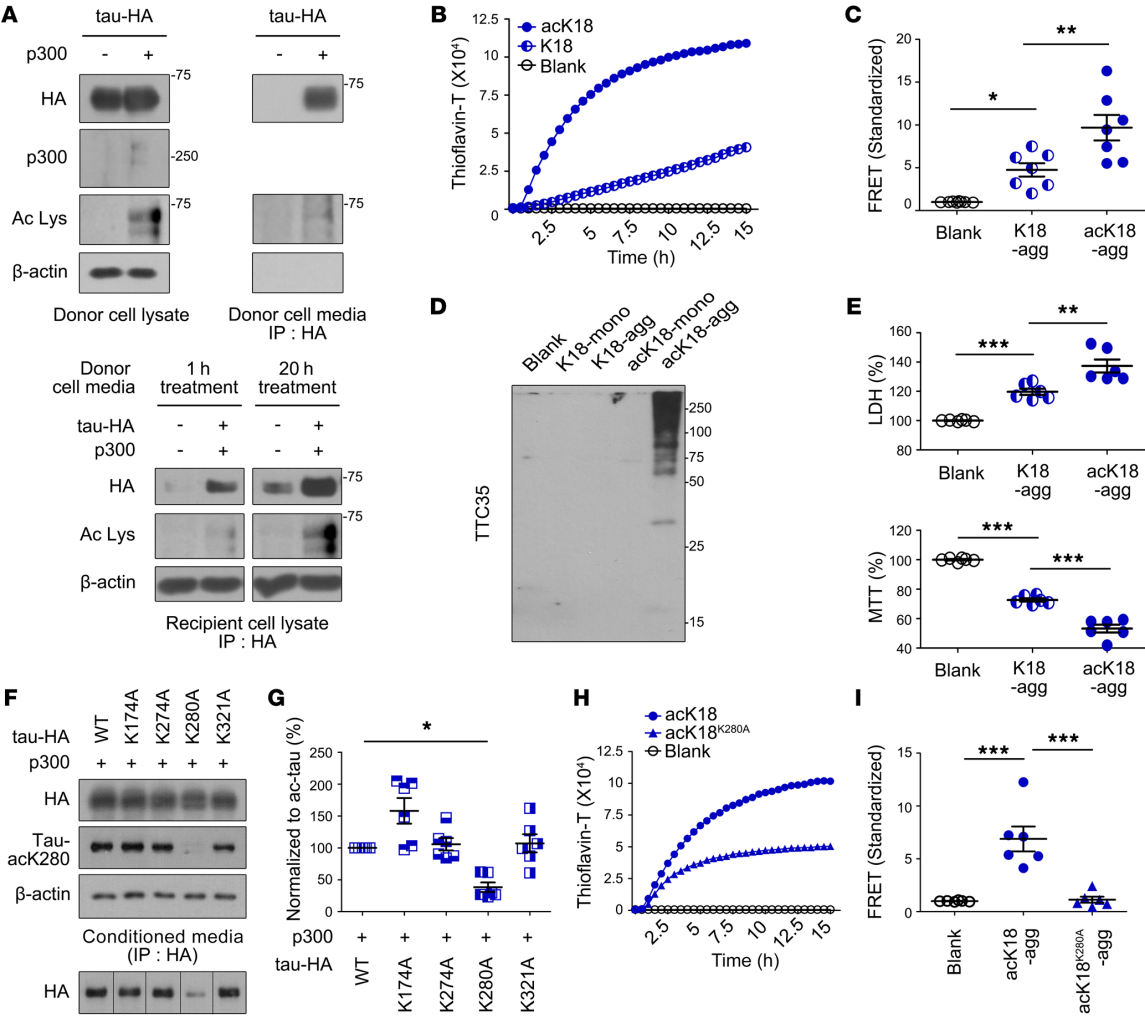
Tau K280 acetylation increases aggregation and propagation. (**A**) Left: Representative immunoblots of tau-HA–expressing donor cell lysates with or without p300 acetyltransferase. Right: Representative immunoblots of culture media from donor cells; immunoprecipitation (IP) was performed with HA antibody. Bottom: Representative immunoblots in recipient cell lysates treated with tau-HA donor cell media for 1 or 20 hours. The lanes in the bottom panel were run on the same gel but were noncontiguous. Ac Lys, anti–acetyl-lysine antibody. (**B**) Tau aggregation profiles determined by thioflavin-T fluorescence using K18 and acK18. (**C**) Tau seeding activity in HEK293T tau biosensor cells treated with K18-agg or acK18-agg, as determined by fluorescence resonance energy transfer (FRET). *n* = 7 per group. (**D**) Tau aggregation in high–molecular weight species. Primary mouse cortical neurons were treated with equal amounts of K18-mono, K18-agg, acK18-mono, or acK18-agg, and tau was detected by semidenatured immunoblotting using TTC35 antibody. (**E**) Representative LDH and MTT assays of primary neurons treated with K18-agg and acK18-agg. *n* = 6 per group. (**F**) Representative immunoblots of donor cells expressing tau-HA (WT, K174A, K274A, K280A, or K321A) acetylated by p300 acetyltransferase. The second row shows representative immunoblots of HA in conditioned media. IP was performed with HA antibody. The lanes at bottom were run on the same gel but were noncontiguous. (**G**) Quantification of tau-HA immunoblots of conditioned media from donor cells. *n* = 6 per group. (**H**) Tau aggregation determined by thioflavin-T using acK18^K280A^ and acK18. (**I**) Tau seeding activity by FRET assay with acK18-agg or acK18^K280A^-agg. *n* = 6 per group. Statistical analysis was performed by 1-way ANOVA followed by Tukey’s multiple-comparison test. *P < 0.05, **P < 0.01, ***P < 0.001. Data are shown as mean ± SEM.

To begin to investigate acetylation-induced tauopathy, we transfected a human neuroblastoma cell line (SH-SY5Y cells) with HA-tagged, full-length WT tau (tau-HA) with or without the histone acetyltransferase p300 (Figure 1A, upper left). In the presence of p300, tau undergoes acetylation at lysine residues (8) and shifts from intracellular localization to extracellular secretion (18). To monitor tau expression profiles, we collected cell culture media from SH-SY5Y cells that expressed either tau-HA alone or both tau-HA and p300, and immunoprecipitated the media with anti-HA antibody. We then performed immunoblots with anti-HA antibody to detect tau secreted from neuronal cells (Figure 1A, upper right). Secreted tau was observed only in culture media from cells expressing both tau-HA and p300, indicating that aberrant tau secretion is dependent on p300-mediated acetylation.

The spread of tau pathology between neurons has been suggested to have devastating effects on the brain (19). We investigated the propagation of acetylated tau (ac-tau) in neuronal SH-SY5Y cells by supplementing native SH-SY5Y (recipient cells) with culture media harvested from SH-SY5Y donor cells expressing either tau and p300 (donor, ac-tau media) or tau only (tau media). After addition of donor media, the recipient neurons were grown for 1 or 20 hours, and the levels of tau in the recipient cells were quantitatively analyzed (Figure 1A, bottom). Surprisingly, lysates from recipient cells treated with ac-tau media contained more tau than lysates from recipient cells treated with tau media. In addition, more tau was observed in recipient cells after 20 hours than after 1 hour. These results demonstrate that acetylation of tau alters the protein in 2 ways: it is secreted extracellularly from neurons and can be transferred into other recipient neurons.

### Tau aggregation and pathology by acetylation.

To observe the acetylation-mediated molecular kinetics of tauopathy, we used the tau fragment K18, which contains 4 MTBRs (corresponding to residues 244–372 of full-length tau, which consists of 441 residues) and is considered a major pathological motif (20). As in the full-length tau study, we prepared acetylated K18 (acK18) by addition of purified p300. Fibrillar aggregation was quantitatively monitored over time by thioflavin-T (ThT) assay (21). As a control, artificial K18 aggregates were prepared by addition of the polyanionic compound heparin to K18 as described previously (20) (Figure 1B and Supplemental Figure 1; supplemental material available online with this article; https://doi.org/10.1172/JCI156537DS1 In comparison with native K18, acK18 exhibited an accelerated and saturable aggregation curve, indicating that tau acetylation triggers fibrillar tau oligomerization, and that aggregation continues until all available tau is exhausted. This is also observed with full-length tau (2N4R) (Supplemental Figure 2).

To determine how acetylation of tau triggers a tauopathy aggregation, we performed single-molecule fluorescence resonance energy transfer (FRET). We tested proteopathic seeding of tau using HEK293T cells stably expressing tau RD P301S-CFP and tau RD P301S-YFP (22). Specifically, we applied K18 aggregates with or without acetylation (acK18-agg and K18-agg, respectively) to the cells, and then monitored tau-stimulated FRET signals. The endotoxin level with acK18-agg was less than 0.1 ng/mL and confirmed that the level did not affect cell viability (Supplemental Figure 3). FRET intensities were 2- to 10-fold higher in cells treated with acK18-agg than in those treated with K18, indicating that intracellular tau proteins came closer to each other when acK18 was added extracellularly (Figure 1C and Supplemental Figure 4). Based on this finding, we hypothesized that tau acetylation initiates tau seeding and stimulates tauopathic aggregation. Because we observed acetylation-mediated seeding kinetics of tauopathic molecules, we wondered whether a similar process occurs in primary neuronal cells. To explore this possibility, we exposed mouse primary cortical neurons (DIV10) to K18 fragments with or without acetylation in monomeric or aggregated form (K18-mono, K18-agg, acK18-mono, or acK18-agg). After 24 hours, we analyzed primary cell lysates by semidenatured Western blotting with the TTC35 antibody, which detects aggregated but not monomeric tau (23). In the blots, primary neuronal cell lysates treated with acK18-agg exhibited a series of high–molecular weight bands (Figure 1D). This suggests that intrinsic tau in primary neuronal cells was transformed into aggregates by acK18-agg. In addition, these observations suggest a possible mechanism for seeding of tauopathy, in which acetylated tau aggregates internalize within recipient neurons and trigger the transformation of soluble intracellular tau into pathogenic aggregates. In order to validate this mechanism further, tau seeding and propagation were performed with 2 successive neuron cultures (Supplemental Figure 5A). K18 fragments with or without acetylation in monomeric or aggregated form were added to the first tau-HA–transfected neurons for 48 hours. Then, the culture medium was completely removed, and neurons were incubated with fresh medium for another 24 hours. This culture medium was transferred to the second recipient neurons. In the first recipient neurons, the upper-shifted smear bands of tau detected with antibody targeting N-terminus (N-term) of tau were most prominent in acK18-agg–treated neurons (Supplemental Figure 5, B and E). This indicates that the tau inside the first recipient neurons itself transformed into pathogenic forms because anti–tau-N-term antibody cannot detect the K18 added to the cultures. Tau was most abundantly released from the first recipient neurons treated with acK18-agg (Supplemental Figure 5, C and F). In the second recipient neurons, the upper-shifted smear bands of tau detected with antibody targeting tau’s microtubule-binding repeating domain (MTBR) were most evident in the neurons treated with the medium of the acK18-agg–treated neurons (Supplemental Figure 5, D and G). These results further support the hypothesis that acetylation plays a key role in tau seeding and propagation.

In tauopathy-linked neurodegenerative diseases, neurons become dysfunctional and vulnerable to cell death (24). We treated primary cortical neurons with either K18-agg or acK18-agg, and then tested neuronal viability by lactate dehydrogenase (LDH) and MTT assays (Figure 1E and Supplemental Figure 6). In the cultures, acK18-agg and K18-agg increased LDH levels and decreased MTT values, indicating that cell membranes and mitochondria were damaged by aggregated K18 species. AcK18-agg increased the LDH level to a greater extent than K18-agg, and decreased the MTT value to a lesser extent, emphasizing that aggregation of the K18 pathological fragment is a primary cause of neuronal cell death and that tau acetylation aggravates neurotoxicity.

### A pathogenic tauopathy caused by tau acetylation at K280.

In neurodegenerative diseases including AD, p300 is phosphorylated and acquires uncontrolled acetylation activity (25, 26). Analysis of tau PTM using ac-tau acetylated in vitro by p300 revealed that 23 lysine residues received acetylation (8). Moreover, in a transgenic *Drosophila melanogaster* expressing a mutant tau, in which K280 residue was replaced by glutamine to mimic the side chain of acetylated lysine, toxic tauopathy effects were observed similar to those caused by acetylated tau (27). In addition to K280, other lysine residues including K174 and K274 are acetylated and contribute to tau fibrillization (28). To delineate the direct effect of tau acetylation on clinical tauopathy in human neurodegenerative diseases, we prepared a series of tau mutants, in which individual lysine residues were replaced by alanine (tau^K174A^, tau^K274A^, tau^K280A^, and tau^K231A^), and expressed them in SH-SY5Y cells with p300 (Figure 1, F and G). We then compared the levels of secreted tau in culture media of cells expressing WT or mutant tau. Among the mutants, tau^K280A^ significantly decreased the amount of extracellular tau (Figure 1F, bottom, and Supplemental Figure 7) without cell death (Supplemental Figure 8). Thus, tau-acK280 induced more tau secretion from neurons than tau acetylated at other lysine residues. This is further supported by the findings that the tau released by p300 was acetylated at K280 (Supplemental Figure 9) and that the expression of an acetylation mimic, K280Q, increased tau release but K280A decreased it (Supplemental Figure 10). Increased release of tau was not observed in primary mouse neurons treated with an acetyltransferase inhibitor (C646), while tau release was increased in neurons treated with a histone deacetylase inhibitor (trichostatin-A [TSA]) (Supplemental Figure 11). Increased acetylation at K280 and its release of endogenous tau were also observed in TSA-treated primary mouse neurons (Supplemental Figure 12).

To ensure that a progressive tauopathy forms via an acK280-mediated aggregation at a molecular level, we introduced a similar mutation in K18, in which a residue corresponding to K280 of tau was mutated to alanine (K18^K280A^). A ThT assay revealed that acK18 aggregation was dramatically reduced in the K18^K280A^ mutant (Figure 1H). Consistent with this, tau seeding FRET assay revealed that aggregation was reduced in K18^K280A^ relative to the K18 WT (Figure 1I). Collectively, these findings indicate that loss of the acetylation site in the K280A mutant alleviated tau secretion and aggregation, confirming that tau-acK280 contributes to progression of pathogenic tauopathy.

### Active immunization with tau-acK280 ameliorates behavioral impairments and pathological changes in tau-transgenic mice.

Based on the pathogenic importance of acK280 (Figure 1), we investigated its therapeutic potential in tau-P301L–transgenic mice. We immunized tau-P301L mice from 3 to 6 months of age with each keyhole limpet hemocyanine–conjugated (KLH-conjugated) tau-acK280 peptide in aluminum adjuvant (Supplemental Figure 13). WT and control tau-P301L mice received adjuvant only. Mice immunized with tau-acK280 demonstrated consistent improvement in cognitive and motor performance, suggesting that tau-acK280 is the effective therapeutic target for AD and tauopathy. Western blot data revealed an increase in abnormally phosphorylated tau in tau-P301L mice, but this effect was decreased after tau-acK280 immunization (Supplemental Figure 14). Acetylated tau was increased in tau-P301L mice, but decreased after tau-acK280 immunization.

### Development of the anti–tau-acK280 antibody, Y01, for study of tauopathy.

**Figure 2:**
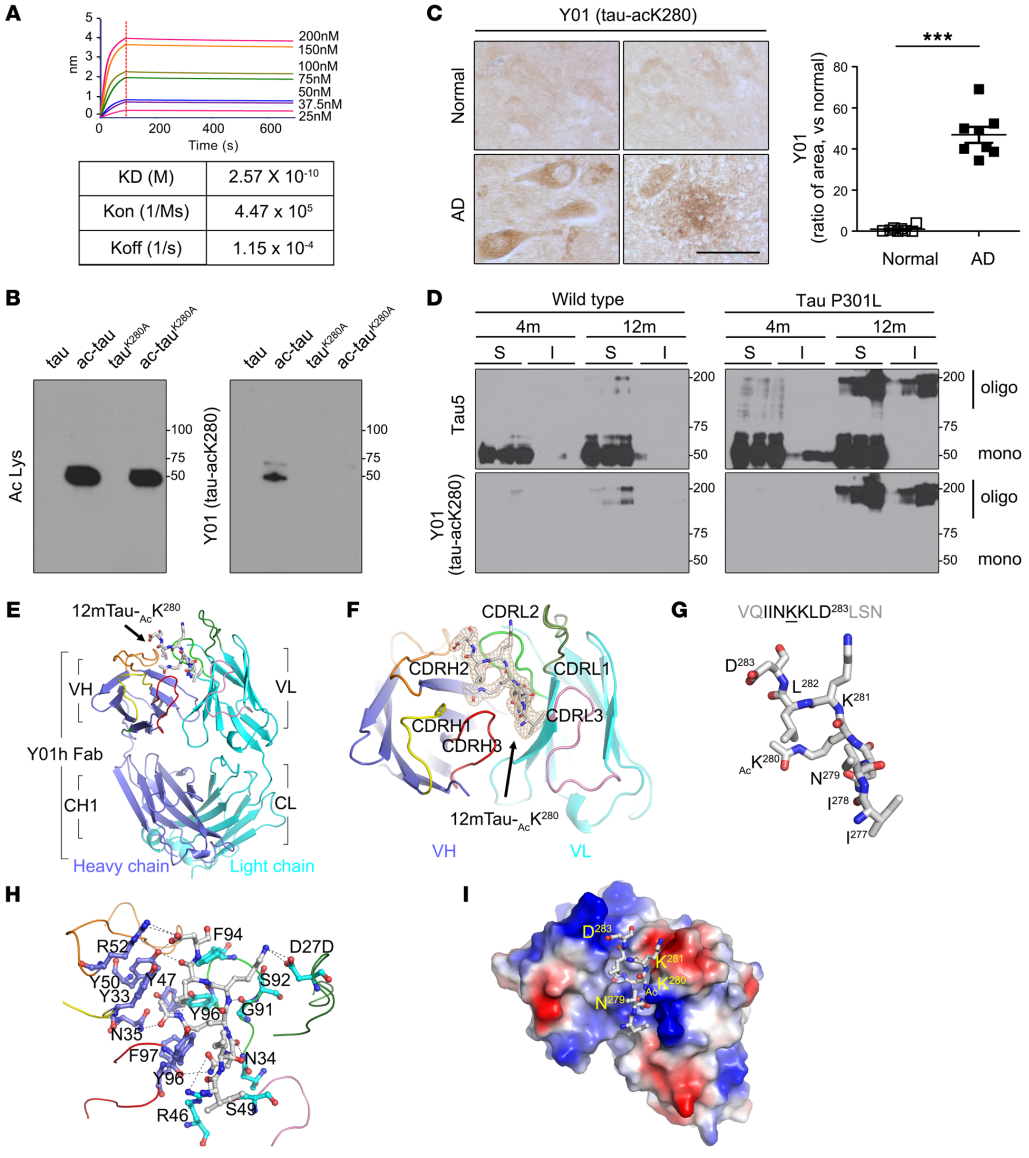
Characteristics and crystal structure of Y01, monoclonal anti–tau-acK280 antibody. (**A**) Specific binding affinity of Y01 for tau-acK280. Dissociation constant between Y01 and ligand was measured by biolayer interferometry (Octet) using the 12mTau-acK280 peptide as the ligand (*K_D_*, dissociation constant; *K*_on_, association rate; *K*_off_, dissociation rate). (**B**) Representative immunoblots of tau proteins (ac-tau, tau^K280A^, and ac-tau^K280A^) with Ac Lys or Y01 antibody. (**C**) Immunohistochemistry (IHC) of normal aged or AD human hippocampus with Y01 antibody. Scale bar: 100 μm. Quantification of Y01 tau pathologies in AD patient hippocampus. *n* = 8 per group. Statistical analysis was performed by 1-way ANOVA followed by Tukey’s multiple-comparison test. ***P < 0.001. The error bars represent the SEM. (**D**) Immunoblots of soluble (S) and insoluble (I) formic acid fractions using mouse brains from 4- and 12-month-old WT and tau-P301L mice. (**E**) Overall complex structure of the Y01 and 12mTau-acK280. (**F**) F_o_–F_c_ electron density map of tau peptide, contoured at 3.0 σ (gray mesh). The difference maps were generated after segmented rigid body, positional, and isotropic B-factor refinements of the antibody in the absence of the tau peptide. (**G**) Sequences and structures of the tau peptide in the complex structure. The Y01 structure is shown as cartoons in subdomain-specific colors (blue, heavy chains; light blue, light chains; yellow, CDRH1; orange, CDRH2; red, CDRH3; light green, CDRL1; green, CDRL2; pink, CDRL3). The tau peptide is shown as gray sticks (gray, carbon; red, oxygen; blue, nitrogen). (**H**) A closed view of the Y01–12mTau-acK280 complex. Y01 is depicted as ribbons. Y01 residues that contact the tau peptide are represented by blue and light blue ball-and-stick models indicating heavy and light chains, respectively (red, oxygen; blue, nitrogen). Hydrogen bonds between Y01 and tau peptide are depicted as broken lines. (**I**) Electrostatic interactions between Y01 and 12mTau-acK280 peptide. The structure of Y01 is shown as an electrostatic potential surface, ranging from acidic (red) to basic (blue). The tau peptide is shown as gray ball-and-stick models (red, oxygen; blue, nitrogen).

Because K280 is critical for acetylation-induced tauopathy (Figure 1) and therapeutic potential of targeting acK280 was observed in vivo in tau-P301L mice (Supplemental Figures 13 and 14), we developed a monoclonal antibody, Y01, that is specific for tau-acK280 and characterized this reagent for use in subsequent analyses. Biolayer interferometry revealed that the dissociation constant (*K_D_*) of Y01 for the acetylated tau peptide 12mTau-acK280 was 2.57 × 10^–10^ M (Figure 2A and Supplemental Figure 15). To ensure the specificity of the Y01 antibody toward tau-acK280, we prepared 2 full-length recombinant tau proteins, 2N4R, and a mutant in which K280 was replaced by alanine, and treated them with p300 to induce in vitro acetylation (Figure 2B). We compared the binding of Y01 to acetylated tau with the binding of another tau antibody, Ac Lys, which was used previously to observe acetylated proteins. As expected, Ac Lys bound both p300-treated tau proteins, 2N4R and 2N4R^K280A^, irrespective of the presence of the K280 residue, as the mutant contained acetylated lysine residues other than K280. Strikingly, Y01 antibody interacted only with p300-treated 2N4R, but not with 2N4R or 2N4R^K280A^, demonstrating selectivity toward tau-acK280.

To confirm that the Y01 antibody could also detect acetylated tau species derived from in vivo samples, we performed immunoprecipitation with Y01 antibody using tau-P301L brain tissues. Western blots revealed that Y01 effectively detected in vivo–acetylated tau from P301L mouse brain (Supplemental Figure 16). Next, we performed immunohistochemistry with the Y01 antibody on hippocampus from human AD patients (Figure 2C and Supplemental Table 2). Using Y01, we could clearly visualize deposition of tau-acK280 in AD neurons, dystrophic neurites, and neuritic plaques (Supplemental Figure 17) similar to AT8 staining pattern. In addition, we tested Y01 to resolve the presence of tau-acK280 in fractionated soluble and insoluble brain samples from 4- and 12-month-old WT and tau-P301L mice (Figure 2D). We detected the most deposition of tau-acK280 in insoluble brain fractions from 12-month-old tau-P301L mice. Therefore, we conclude that the Y01 antibody has a specific binding affinity for tau species containing acK280 residue regardless of antigenic tau origins, such as synthetic peptide, purified recombinant protein, or in vivo brain samples. Since therapeutic antibody should target an extracellular antigen, we wondered whether tau-acK280 exists in human extracellular space and can be targeted by Y01. Hence, we performed dot blots with Y01 using human cerebrospinal fluid samples (Supplemental Figure 18) and found the extracellular presence of tau-acK280 in human samples and its detection by Y01.

### Crystal structure of the anti–tau-acK280 antibody Y01.

The selective binding of Y01 to acetylated tau in a K280-dependent manner motivated us to determine the structure of the Y01 Fab. We determined the crystal structure of the Y01 and 12-mer tau^K280^ complex by molecular replacement, and refined it to *R*_work_ and *R*_free_ values of 19.51% and 24.52%, respectively, at a resolution of 2.5 Å (Figure 2, E–G, and Supplemental Table 3). In the complex structure, 7 residues of 12-mer tau-acK280, corresponding to tau residues 277–283, were modeled with good refinement statistics (Figure 2, F and G). To our knowledge, this is the first reported structure in which the acetyl atoms of acK280 can be directly observed in a crystal structure in complex with an antibody.

The complex structure revealed that the Y01 epitope recognition site forms at a groove consisting of complementarity-determining region (CDR) residues of VH and VL domains, where the tau-acK280 peptide binds. At the rim of the Y01 groove, the peptide backbones of the 12-mer tau-acK280 are clipped by a network of hydrogen bonds with Y01 residues (Figure 2H). The carbonyl oxygen atoms of I277, N289, and L282 of the 12-mer tau-acK280 contact R46 and N34 from the LH domain and Y47 from the VH domain, respectively. At K281 of the tau peptide (tau^K281^), the main chain nitrogen atom forms a hydrogen bond with the VH Y50 residue of Y01. In addition, the electrostatic surface potential map of the Y01 Fab revealed chemical complementarities between polar tau residues at tau^N279^, tau^K281^, and tau^D283^ and Y01 residues R46 and D27D of the LV domain and R52 of the VH domain, respectively, indicating the residue specificity of Y01 epitope recognition (Figure 2I). Overall, the complex structure emphasizes that the side chain atoms of acK280 are deeply plugged into the Y01 antigen binding site. Four aromatic side chains — Y47, Y96, and F97 from the VH domain and Y96 from the LH domain — surround the aliphatic side chain atoms of tau^K280^, and the VH N35 nitrogen atom forms a hydrogen bond with the acetyl oxygen atom of tau^K280^. Therefore, the structure of the Y01–tau peptide complex reveals that Y01 specifically recognizes acK280 and its surrounding residues, and suggests that their direct interaction potentiates the neutralizing activity toward tauopathy caused by tau-acK280.

### Y01 inhibits acetylation-induced tau aggregation and propagation, and promotes microglial tau phagocytosis.

**Figure 3:**
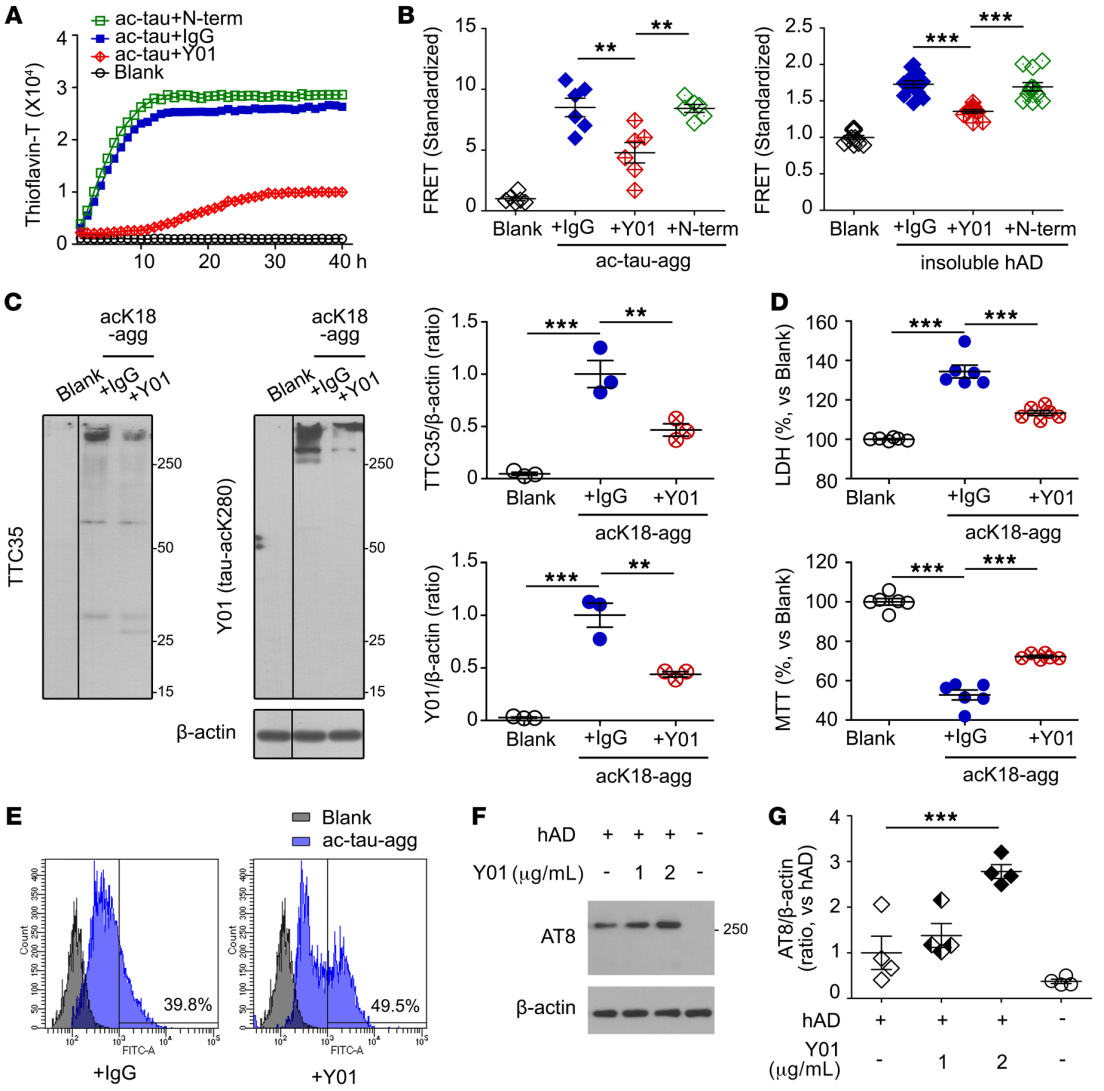
Y01 antibody inhibits acetylation-induced tau aggregation and propagation but enhances microglial tau uptake by Y01. (**A**) Y01 antibody decreases aggregation of acetylated tau. IgG was used as a control. Anti–tau-N-term antibody does not decrease aggregation of acetylated tau. (**B**) Quantification of Y01-mediated inhibition of the FRET signal from HEK293T tau biosensor cells. Cells were pretreated with either Y01, anti–tau-N-term, or IgG, and tau seeding was induced by addition of either ac-tau-agg (*n* = 6 per group) or sarkosyl-insoluble fractions from human AD brain (*n* = 12 per group). (**C**) Y01 antibody decreased tau aggregation in primary mouse cortical neurons treated with acK18-agg. IgG was used as a control. The lanes were run on the same gel but were noncontiguous. AcK18-agg was analyzed by semidenatured immunoblotting using TTC35 or Y01 antibody, and aggregation was quantified by densitometry. *n* = 3 per condition. (**D**) Representative LDH and MTT assays of primary neurons treated with acK18-agg and control IgG or Y01. *n* = 6 per group. (**E**) Flow cytometric quantification of mean fluorescence intensity (arbitrary units) of primary mouse microglia treated with ac-tau-agg in the presence of control IgG or Y01. (**F** and **G**) BV2 microglial cells were treated with sarkosyl-soluble fractions from AD brains with either control IgG (2 μg/mL) or Y01 (1 or 2 μg/mL) for 4 hours. (**F**) Semidenaturing immunoblots with AT8 antibody. (**G**) Quantification of AT8 levels normalized to β-actin. *n* = 4 per group. Statistical analysis was performed by 1-way ANOVA followed by Tukey’s multiple-comparison test. **P < 0.01, ***P < 0.001. The error bars represent the SEM.

The observed specific binding of Y01 to tau-acK280 suggests that this antibody could be used in clinical applications to treat diseases caused by tauopathies mediated by K280 acetylation. To test this idea, we examined the effects of Y01 on a series of events involved in the progression of tauopathy: aggregation, seeding, and propagation. Strikingly, in a ThT assay, addition of Y01 dramatically decreased aggregation of p300-treated tau in a concentration-dependent manner, whereas IgG had no effect (Figure 3A and Supplemental Figure 19, A and B). In FRET experiments, Y01 prevented cellular tau from aggregation induced by acetylated tau aggregates (ac-tau-agg) (Figure 3B). Y01 also inhibited seeding of tau aggregation in the sarkosyl-insoluble fraction of human AD brain (Figure 3B, right; Supplemental Figure 19C; and Supplemental Table 2). These results indicate that the Y01 antibody may have the potential to reduce the progression of tauopathy aggregation and seeding induced by in vitro p300 acetylation, as well as by in vivo human AD tau aggregates. We also compared these anti-aggregation and anti-seeding effects of Y01 with the effects of anti–tau-N-term antibody (Figure 3, A and B). Anti–tau-N-term antibody could not inhibit the aggregation of acetylated tau or the seeding induced by acK18-agg or by the sarkosyl-insoluble fraction of AD brain.

Next, we investigated the effect of Y01 on tauopathy propagation at the cellular level. In neurodegenerative diseases including AD, tauopathy spreads through neurons until it damages the entire brain. We supplemented primary cortical neurons with Y01 and tested them for any change in tau species by monitoring the level of acK18-agg. Semidenatured Western blotting revealed that the levels of smear bands corresponding to tau aggregates were substantially lower in primary neurons treated with Y01 than in neurons treated with IgG (Figure 3C). In addition, we monitored the viability of neurons treated with Y01 or IgG. We treated primary neurons with acK18-agg with either IgG or Y01, and assessed cellular viability by LDH and MTT assays. Relative to pretreatment with IgG, pretreatment of primary neurons with Y01 decreased LDH and slightly increased MTT values (Figure 3D). Normal neuronal microtubule structures were not affected by Y01 itself (Supplemental Figure 20). These data suggest that Y01 exerts a beneficial effect on neuronal survival by decreasing the propagation of tauopathy.

To prevent the propagation of tauopathy in neurons, it would be desirable to remove tau aggregates. Because Y01 is specific for acK280, it should be possible to induce antibody-mediated phagocytosis upon interaction of Y01 and tau-acK280. We tested this idea by treating mouse primary microglia with acetylated tau aggregates and either Y01 or IgG, and then assessed the levels of phagocytosed tau by flow cytometry. When the cells were treated with Y01, larger amounts of acetylated tau aggregates were phagocytosed in microglia than when the cells were treated with IgG (Figure 3E). This demonstrates that Y01 also promotes microglial clearance of acetylated tau aggregates through direct interactions with tau-acK280. This is further supported by an experiment with human sarkosyl-soluble fraction (Supplemental Figure 21A, S2). The microglial cell line BV2 was treated with AD sarkosyl-soluble fraction with either control IgG or Y01, and the increased tau uptake by Y01 was observed (Figure 3, F and G).

### Systemic administration of Y01 antibody ameliorates behavioral deficits and pathological changes in tau-Tg mice.

**Figure 4:**
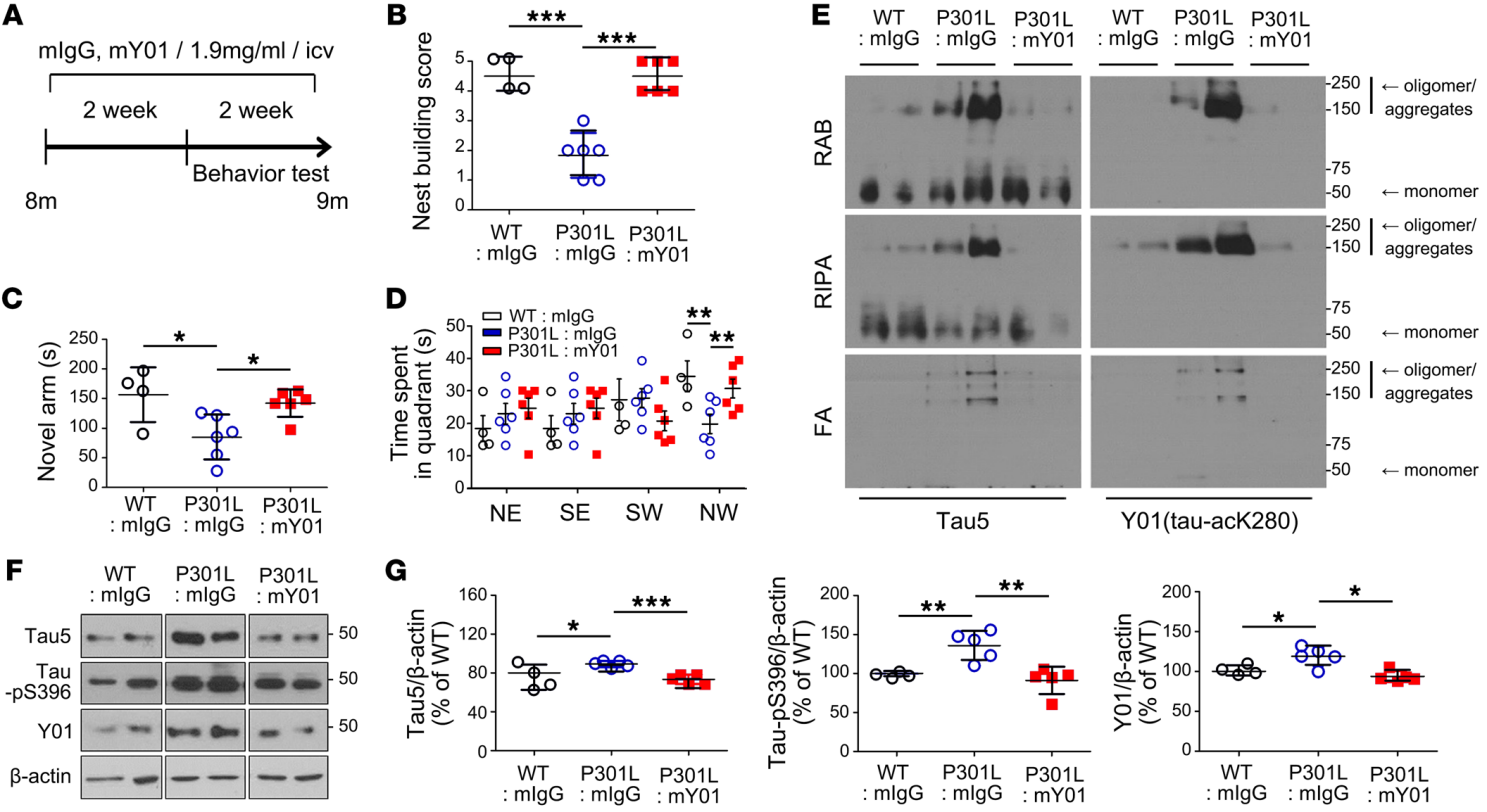
Amelioration of cognitive deficits and pathology by i.c.v. infusion of mouse-Y01. (**A**) Schematic diagram of the passive immunization experimental design. (**B**–**D**) Two weeks after the start of infusions, behavioral tests were performed during the next 2 weeks of infusion. (**B**) Nest building test. (**C**) Y maze. (**D**) Morris water maze. WT + IgG, *n* = 4; tau-P301L + IgG, *n* = 6; tau-P301L + Y01, *n* = 6; all mice were male. NE, SE, SW, and NW indicate northeast, southeast, southwest, and northwest quadrants. Target quadrant: NW. (**E**) Immunoblots of Tau5 and Y01 in RAB fractions, RIPA fractions, and formic acid (FA) fractions of the cortex from immunized mice. WT + IgG, tau-P301L + IgG, tau-P301L + Y01, *n* = 2 per group; all mice were male. (**F**) Immunoblots of Tau5, tau-pS396, and Y01 in the cortex from immunized mice. The lanes were run on the same gel but were noncontiguous. (**G**) Quantification of Tau5, tau-pS396, and Y01 protein levels normalized to β-actin. WT + IgG, *n* = 4; tau-P301L + IgG, *n* = 5; tau-P301L + Y01, *n* = 5; all mice were male. Statistical analysis was performed by 1-way ANOVA followed by Tukey’s multiple-comparison test. *P < 0.05, **P < 0.01, ***P < 0.001. The error bars represent the SEM.

**Figure 5:**
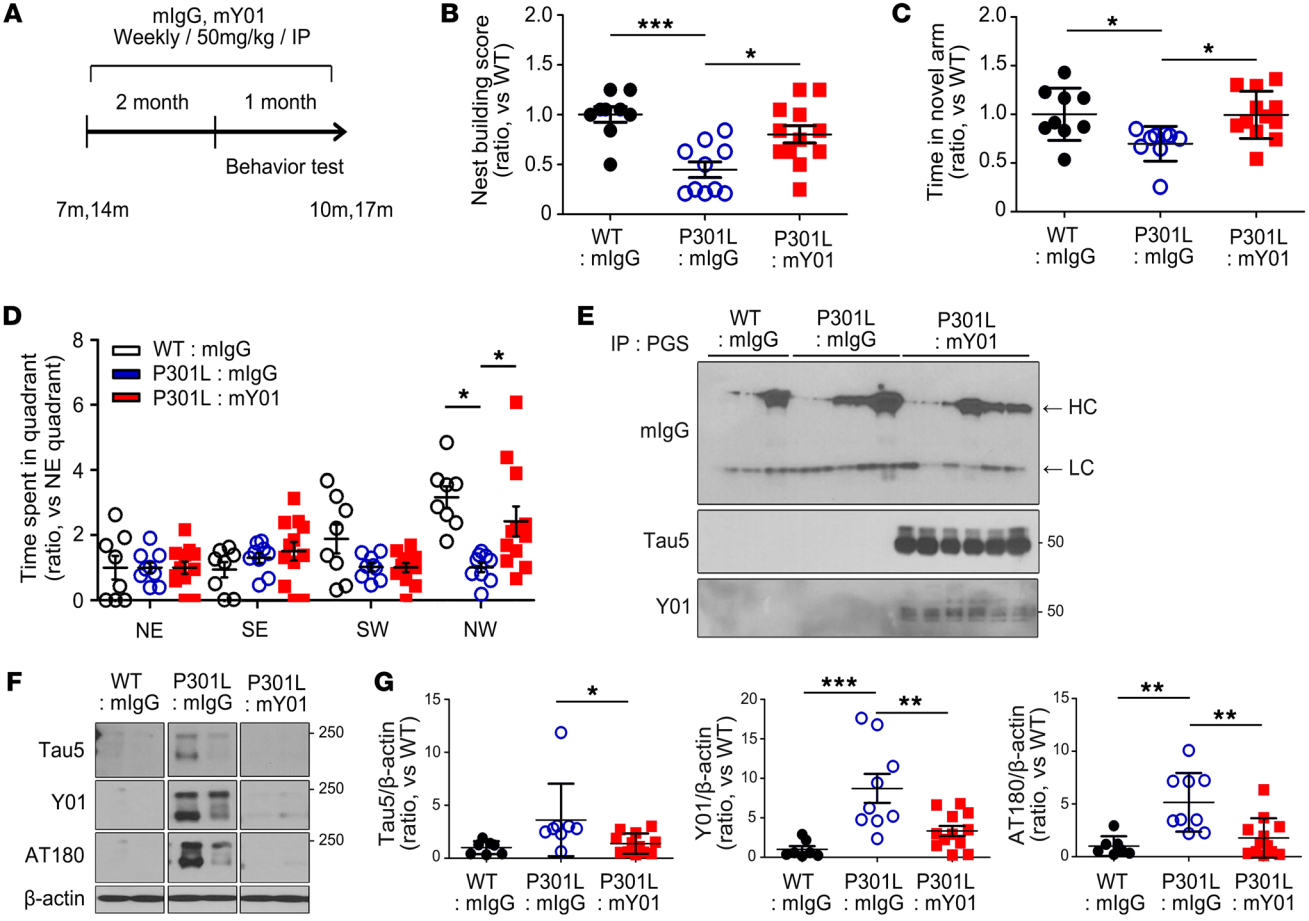
Amelioration of cognitive deficits and pathology by i.p. injection of Y01. (**A**) Schematic diagram of the passive immunization experimental design (age 7 or 14 months). (**B**–**D**) After weekly injections of control mouse IgG or mouse Y01 during 2 months, behavioral tests were performed during the next 1 month of weekly injections. (**B**) Nest building test. (**C**) Y maze. (**D**) Morris water maze. Ages 7 and 14 months; WT + IgG, *n* = 9; tau-P301L + IgG, *n* = 10; tau-P301L + Y01, *n* = 12. NE, SE, SW, and NW indicate northeast, southeast, southwest, and northwest quadrants. Target quadrant: NW. (**E**) Representative immunoblots of mouse IgG, Tau5, and mouse Y01 proteins coimmunoprecipitated with the protein G–Sepharose (PGS) from the IgG- or Y01-injected mouse cortex. HC arrow, heavy chain; LC arrow, light chain. Age 7 months; WT + mIgG, *n* = 3; tau-P301L + mIgG, *n* = 5; tau-P301L + mY01, *n* = 6. (**F**) Representative semidenatured immunoblots of Tau5, Y01, and AT180 protein levels. The lanes were run on the same gel but were noncontiguous. Age 14 months; WT + mIgG, *n* = 4; tau-P301L + mIgG, *n* = 4; tau-P301L + mY01, *n* = 6. (**G**) Quantification of Tau5, Y01, and AT180 protein levels normalized to β-actin. Ages 7 and 14 months; WT + mIgG, *n* = 7; tau-P301L + mIgG, *n* = 9; tau-P301L + mY01, *n* = 12. Statistical analysis was performed by 1-way ANOVA followed by Tukey’s multiple-comparison test. *P < 0.05, **P < 0.01, ***P < 0.001. The error bars represent the SEM.

Y01 inhibits tauopathy and promotes neuronal survival via direct and specific recognition of acetylated tau at K280 (Figures 2 and 3). Using a preclinical disease model in mice, we investigated the potential use of Y01 as a therapeutic. To assess Y01’s therapeutic potential, the intracerebroventricular (i.c.v.) infusion of the murine version of Y01 (mY01) into the lateral ventricle of tau-P301L mice (Figure 4) or the intraperitoneal (i.p.) injection of mY01 (Figure 5) was performed according to the depicted schedules. Improvements in behavioral tests, including nest building test, Y maze, and Morris water maze test (Figures 4 and 5), were observed following administration of Y01 antibody. To investigate whether the i.p.-injected antibody entered the brain and bound to antigens, brain samples were perfused systemically with cold PBS at sacrifice to remove any blood from the brain and were then incubated with protein G–Sepharose (PGS) to pull down the antibodies in brain parenchyma. Western blots with anti-mouse IgG antibody revealed that both control IgG and Y01 antibody were present in the brain (7 months aged; Figure 5E), confirming that antibodies entered the brain even though the blood-to-brain ratio may have been low. Tau protein acetylated at K280 was also pulled down alongside PGS only in Y01 antibody–injected mouse brains, indicating that peripherally injected Y01 antibody entered the brain and bound to the antigen, tau-acK280. Semidenatured Western blots with cortex lysates from mice 17 months of age revealed that total tau (Tau5), tau-acK280 (Y01), and tau-pT231 (AT180) aggregate levels were decreased in antibody-injected brains (Figure 5F). To further evaluate the efficacy of Y01 in a different tau-Tg strain, Y01 antibody was injected i.p. into tau-P301S mice at 2 different doses (5 mg/kg and 50 mg/kg) for 3 months starting from 5 months of age (Supplemental Figure 22). Improvements in the water maze test and synaptic integrity were observed in the group that was given 50 mg/kg of Y01 (Supplemental Figure 22B and Supplemental Figure 23). Semidenatured Western blots with hippocampus lysates that were fractionated into formic acid from 8 months of age revealed that levels of total tau (Tau5) and tau-acK280 (Y01) monomers and oligomers/aggregates were decreased in the brains of mice injected with 50 mg/kg of Y01 (Supplemental Figure 22, C and D).

### Y01 prevents in vivo seeding and propagation of human AD-derived tauopathy.

**Figure 6:**
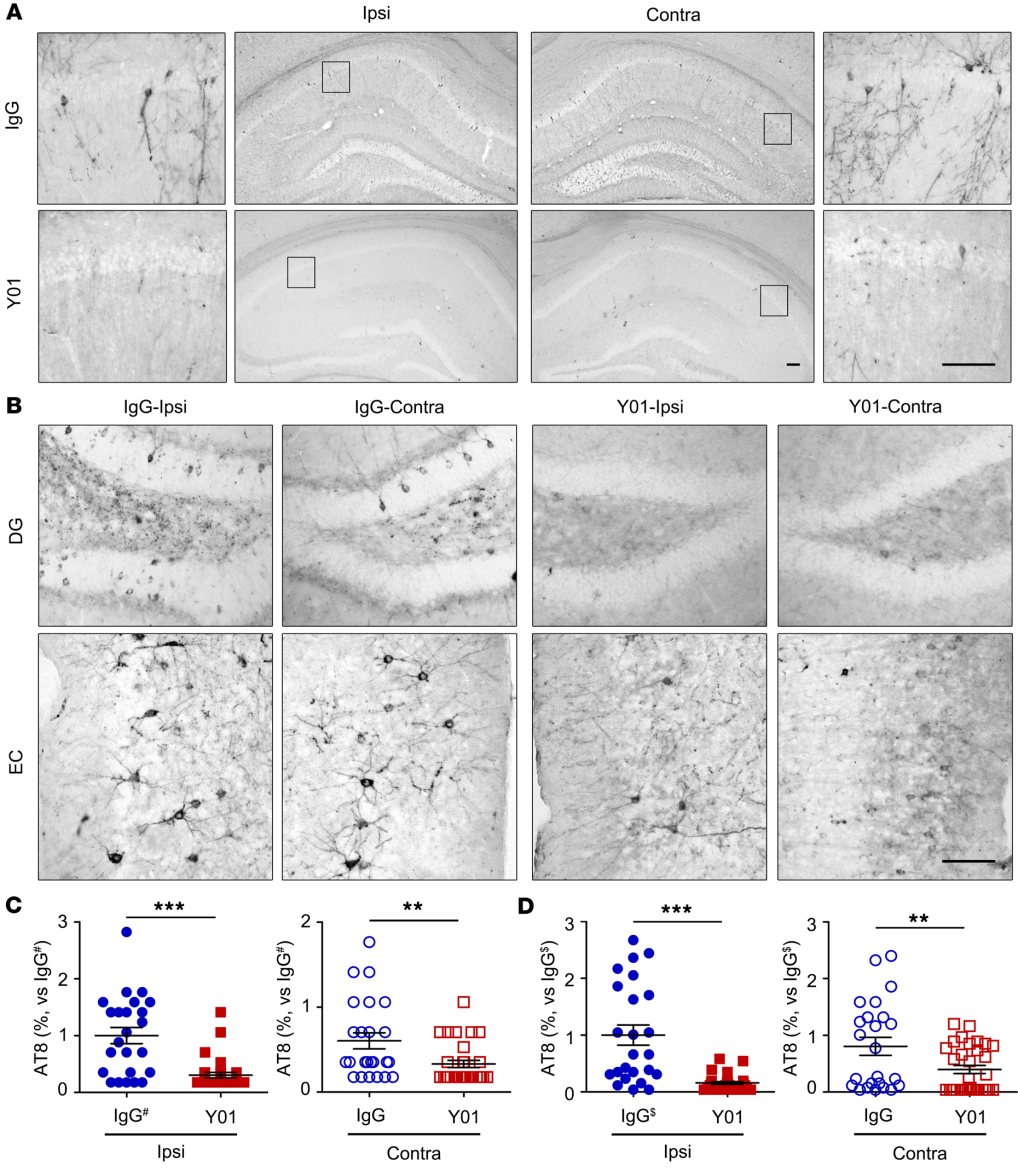
Inhibitory effects of Y01 antibody on tau seeding and propagation in vivo. Four-month-old tau-P301S mice were stereotaxically injected in the left CA1 layer with sarkosyl-insoluble tau fractions from AD patients. Either control mouse IgG or mouse Y01 was administered via intravenous injection once a week for 3 months, and brains were analyzed by IHC analysis using tau AT8 antibody. (**A** and **B**) IHC images of ipsilateral or contralateral hippocampus (**A**) and ipsilateral or contralateral dentate gyrus (DG) or entorhinal cortex (EC) (**B**). Scale bars: 100 μm. (**C** and **D**) Numbers of AT8-positive cells in the ipsi- (left) and contralateral (right) hippocampus and EC layers. # indicates AT8 AT8 quantification of IgG-injected ipsilateral side of dentate gyrus. $ indicates AT8 quantification of IgG-injected ipsilateral side of entorhinal cortex. Analysis in **C** were normalized with IgG^#^ of ipsi. Analysis in **D** were normalized with IgG^$^ of ipsi. Unpaired *t* test; tau-P301S + IgG, *n* = 6; tau-P301S + Y01, *n* = 8. **P < 0.01, ***P < 0.001. The error bars represent the SEM.

The sarkosyl-insoluble fraction from human AD brain (Supplemental Figure 21, P2) was introduced into the left hippocampus CA1 region of 4-month-old P301S-Tg mice using the stereotaxic injection system. The murine version of Y01 (mY01) or control IgG (20 mg/kg) was intravenously administered weekly for 12 weeks. In mice treated with IgG, the number of AT8-positive neurons containing accumulations of tau increased in both the ipsilateral and the contralateral hippocampus (Figure 6A), indicating neuronal damage via ipsilateral seeding and contralateral propagation by tauopathy. By contrast, administration of mY01 dramatically decreased the number of AT8-positive neurons containing tau accumulation in the dentate gyrus (Figure 6, B and C) of hippocampus and entorhinal cortex (Figure 6, B and D). Therefore, we conclude that treatment with the tau-acK280–targeting antibody mY01 in this mouse model exerts a beneficial inhibitory effect on proteopathic seeding induced by AD-derived insoluble tau aggregates and propagation both in vivo and in vitro. In this context, GFAP-positive reactive astrocytes were decreased in the hippocampus of mY01-injected mice (Supplemental Figure 24A), suggesting the alleviation of AD fraction-induced inflammation by Y01. Interestingly, AT8-positive microglia were observed in Y01-injected hippocampus (Supplemental Figure 24B), showing a snapshot of microglia phagocytosing tau.

## Discussion

The hexapeptide ^275^VQIINK^280^, encompassing the K280 residue of tau, has been proposed to be critical for tau aggregation (29), and acetylation of tau at K280 (tau-acK280) has been reported in tauopathies associated with AD and other neurodegenerative diseases (30). However, the role of tau-acK280 in tau secretion and propagation has not previously been elucidated at the molecular and cellular levels. In this study, we performed extensive investigations to show that tau acetylation at K280 is essential for propagation of tauopathy, leading to progression of irreversible neurodegenerative diseases such as AD. We generated the antibody Y01, which targets tau at acK280, and investigated its potential as a therapeutic for neurodegeneration associated with acK280 tau–mediated tauopathy.

Tau acetylation is a key pathogenic PTM that decreases binding to microtubules, increases tau aggregation, decreases tau degradation, and ultimately leads to synaptic dysfunction and loss (8, 16, 17). Acetylation of tau at K274 (acK274) in the first repeat region of the MTBR has been implicated in synaptic dysfunction and memory deficits in AD (17). Acetylation of tau at another residue, K174 (acK174) in the proline-rich region, has been detected in early-stage AD brains, and is associated with malfunctioning tau protein and cognitive deficits (31). Because removal of acetylated lysine at tau 174 by mutation to arginine does not prevent tau-mediated neurodegeneration, an additional acetylation site was proposed to exist (31). Recently, tau-acK280 was reported in multiple types of neurodegenerations and attracted particular interest. Patients with frontotemporal dementia and parkinsonism linked to chromosome 17 (FTDP-17) lack the K280 residue and develop tau deposits in the brain (32). Thus, absence or alteration of tau K280 induces tauopathies in neurodegenerative disease. Such observations emphasize that tau protein must retain K280 to perform its native functions in neurons; otherwise, tau irreversibly transforms into proteopathic tau. Upon acetylation at K280, tau becomes vulnerable to the development of tauopathy in AD and other neurodegenerative disorders (30). Tau-acK280 tends to produce short fibrillar aggregates shorter than 200 nm that may increase the seeding capacity and toxicity of tau species in vivo (33). However, unlike most known tau acetylation sites implicated in AD pathogenicity, tau acetylation at K321 (acK321) inhibits tau aggregation and induces phosphorylation at serine 324 (34). Therefore, not all tau acetylation mediates tauopathy, and acetylation at different sites in the protein exerts distinct effects on tau pathogenicity (31).

Using mutant tau proteins and peptides, we demonstrated that tau acetylation triggers extracellular secretion of tau from neurons and can thus be considered as an initiating cause of tau propagation (Figure 1). To understand tau-mediated tauopathy, it is critical to elucidate the process by which tau transforms into tauopathic states. Several mechanisms have been proposed, but the initial cue responsible for tau secretion has not been explained in detail (35). Inside neurons, chaperone-mediated autophagy (CMA) removes cellular waste, but CMA is impaired by acetylated tau oligomers, which are then located toward late endosome for tau secretion and propagation (36). During CMA-mediated degradation, tau binds the chaperone HSC70 in a pH-dependent manner, but this interaction is abolished by the tau^K280Q^ mutant (36), in which glutamine mimics an acetylated lysine; this observation suggests that tau-acK280 impairs removal of proteinaceous aggregates. Moreover, internal tau is acetylated at K280 upon addition of exogenous tau aggregates, accelerating tau aggregation and prion-like templating concomitant with inhibition of histone deacetylase-6 (HDAC6) (37). Based on these findings, we conclude that acK280 is a critical initial factor in tau secretion (Figure 1), which is influenced by acetylation-mediated aggregation and phagocytic inhibition.

We assessed proteopathic tau seeding and propagation in tau-FRET assays and in vivo stereotaxic seeding and propagation experiments using AD brain–derived insoluble tau aggregates (Figures 3 and 6). These processes were effectively inhibited by Y01, an acK280-targeting antibody, suggesting that tau-acK280 is the core seeding-competent species in the AD brain. Several therapeutic anti-tau antibodies tested in clinical trials target the N-term of tau, as N-terminally truncated forms have been observed in the extracellular space and could provide sources of propagation (38). However, this idea has been questioned by other researchers who argue that the antibody must attack the mid-region of tau to block its pathogenic spread (39). The UCB antibody targets amino acids 235–250, which lie at the end of tau’s second proline-rich region, just before the MTBR. The Janssen antibody also targets the mid-region of tau. These antibodies efficiently stop the seeding and propagation of AD-derived tau, whereas antibodies against the N-term inhibit seeding only very weakly (39), emphasizing the importance of selecting the right epitopes to achieve therapeutic effects. This conclusion is also supported by very recent failures of anti-tau antibodies targeting N-term in AD clinical trials. Most antibodies in clinical trials target unmodified forms of tau (https://www.alzforum.org/therapeutics) based on the assumption that all extracellular tau is pathogenic, regardless of modifications. However, accumulating evidence demonstrates that full-length or truncated forms of tau are also present under normal conditions in the extracellular space, such as in cell culture media, interstitial fluid (ISF), and cerebrospinal fluid (CSF) (40). The physiological function of extracellular tau remains unclear, and it is not clear which forms of tau are physiological or pathogenic. Accordingly, it is important to ensure that therapeutic tau antibodies do not target the physiological forms of tau.

By targeting tau-acK280, the Y01 antibody inhibits tau aggregation and propagation, and promotes microglial tau clearance (Figure 3). Aggregated forms of tau are prone to proteopathic seeding and transcellular propagation of tau. It is also possible that tau aggregates in the extracellular space, where it is converted into pathogenic forms, but this has not been proven (41). Hence, a preferred mechanism of action for therapeutic tau antibodies would be to target the extracellularly released aggregation-prone forms of tau to directly inhibit tau aggregation outside the cell. Tau acetylation at K280 is critical for aggregation and secretion of tau (Figure 1), and induces formation of insoluble aggregates triggered by hydrophobic pockets (15). Upon acetylation, the chemical and spatial properties of positively charged K280 are altered by acquisition of a negatively charged oxygen and an extended hydrophobic methyl group. Moreover, acetylation of K280 would simultaneously induce a conformational shift at adjacent residues, including N279, K281, L282, and K283, leading to aggregation. However, aggregation induced by acetylation at K280 could be neutralized by an addition of Y01. Indeed, the crystal structure of the complex of Y01 and acetylated tau peptide revealed that Y01 directly recognized the acetyl-lysine side chain atoms of tau K280 and its surrounding residues. By specifically targeting acK280 as well as stabilizing adjacent residues, Y01 can prevent parallel stacking of tau filaments and thereby halt the progressive destructive accumulation of tau aggregates. In addition, addition of Y01 restored phagocytic removal of aggregated tau through antibody-mediated endocytosis. Collectively, these observations indicate that the beneficial effect of Y01 in tauopathy is due to Y01-mediated neutralization and endocytosis of tau-acK280.

It is important to note that preclinical efficacy studies using transgenic mouse models have inherent limitations. Since tau-Tg mouse models like PS19 are highly variable in phenotypes (42) and sex is a major confounding factor, Sun et al. recommended the use of male tau-Tg mice for evaluating the effect of therapeutic antibody (43). Hence, researchers tried to use only male mice in the efficacy studies (Supplemental Table 4), which may limit application to human disease conditions. Y01 has a pharmacokinetic profile similar to those of other antibodies such as BIIB076 (44). Y01’s half-life was 185 hours and its blood-brain barrier permeability 0.275% in Sprague-Dawley rats (Supplemental Figure 25). Total tau in the CSF or ISF is at very low levels of picomolar range (45, 46); hence tau-acK280 must be at lower levels of picomolar or even femtomolar range. Assuming the concentration of drug is far greater than the concentration of target, like nanomolar range of antibody (47) and pico- or femtomolar range of target, tau-acK280, target occupancy could be calculated from the Michaelis-Menten–style hyperbolic equation: occupancy = [mAb]/(*K_D_* + [mAb]), where *K_D_* is the dissociation constant (48). At 5 nM and 2 nM of Y01 (*K_D_* = 257 pM), target occupancy is calculated as 95.1% and 88.6%, respectively, from which we expect Y01 could achieve sufficient target engagement. Since this calculation is based on the avidity of Y01 and not on the monovalent affinity, the target occupancy may be overestimated, and future elaborative studies are necessary for the exact measurement and calculation (49). Interestingly, the relative level of CSF tau-acK280 was decreased in mY01-injected mice (Supplemental Figure 26), showing pharmacodynamics of antigen-antibody binding. Since this measurement was made with Y01-coated ELISA as described in Supplemental Methods, it could reflect the decrease of Y01-unbound tau-acK280, suggesting target engagement. However, total tau-acK280 could also be decreased, possibly by the mechanism of microglia-mediated degradation or inhibition of tau accumulation.

Finally, the findings in this study indicate that tau-acK280 is a therapeutic target of the Y01 antibody, which inhibits tau aggregation, secretion, and propagation. Thus, Y01 represents a promising new therapeutic antibody for AD and other tauopathies.

## Methods

### Animal models

Studies were performed in C57BL/6J-WT, tau-P301L, and tau-P301S–transgenic mice. Tau-P301L mice were generated from JNPL3 (tau-0N4R, P301L) mice purchased from Taconic Inc. and backcrossed to C57BL/6J over 5 generations. Tau-P301S mice were generated from PS19 (tau-1N4R, P301S) mice purchased from The Jackson Laboratory and backcrossed to C57BL/6J over 5 generations. All mouse genotyping was confirmed by PCR. Information on the sexes of mice used in the study is summarized in Supplemental Table 4. All animals (both sexes) were housed on a 12-hour light/dark cycle at constant ambient temperature (22°C ± 1°C) with 40%–60% humidity. Food and water were provided ad libitum in a specific pathogen–free animal facility (3–5 mice per cage).

### Tau acetylation and filament assembly

To acetylate tau, 8 μM recombinant tau protein (full-length tau, K18, or K18^K280A^) was mixed with 0.5 μg purified GST-p300 and 125 μM acetyl-CoA (Sigma-Aldrich) in acetylation buffer (10 mM HEPES, 50 mM NaCl, 1.5 mM MgCl_2_, 0.5 mM DTT, 2.5 mM EGTA, 0.1 mM EDTA) and incubated for 3 hours at 30°C with agitation at 300 rpm (Eppendorf Thermomixer C) (34). To prepare aggregated tau species, 25 μM tau protein in monomeric soluble form was incubated in aggregation buffer (2 mM MgCl_2_, 1 mM DTT, and 25 μM heparin) at 37°C with agitation (50, 51). For the phagocytosis assay, Alexa Fluor 488–labeled, acetylated tau aggregates were prepared from tau monomer fluorescently labeled using the HiLyte Fluor 488 Protein Labeling Kit (AnaSpec, AS-72047) and then were subjected to acetylation and aggregation.

### Human samples

Paraffin brain tissue slides from AD patients and control brain from healthy non-demented individuals were obtained from the Netherlands Brain Bank. Blinded frozen brain tissues and postmortem cerebrospinal fluid (CSF) were provided by the Korea Brain Bank, and human CSF was provided by the Samsung Medical Center with Asan Medical Center IRB approval (2018-1174). The demographic characteristics of the subjects are listed in Supplemental Table 2. All experimental procedures were carried out in accordance with the relevant guidelines, laws, and regulations.

### Transient transfections

SH-SY5Y cells were cultured in DMEM (high glucose, HyClone) supplemented with 10% FBS and 1% antibiotics at 37°C and 5% CO_2_. Cells were seeded at 2.5 × 10^5^ cells per well on 12-well plates. MAPT (microtubule-associated protein tau [*Homo sapiens*, human]; tau) plasmid was transfected with or without EP300 (E1A binding protein p300 [*Homo sapiens*, human]; p300) plasmid using Lipofectamine 2000 (Thermo Fisher Scientific) in Opti-MEM (Gibco). After 24 hours of transfection, the supernatant was harvested for further analysis by immunoprecipitation or for transfer to the recipient cells.

### ThT assay

Tau fibrillization was achieved by incubation of 10–20 μM tau protein and ThT solution at a 1:1 ratio in a total 40 μL aggregation buffer with 0.5 mg/mL heparin at 37°C with agitation at 300 rpm, as previously described (50, 51). ThT fluorescence was measured at various time points using CLARIOstar (BMG Labtech) in a 384-well plate (Greiner Bio-One). Detailed materials and methods are described in Supplemental Methods.

### FRET P301S tau biosensor cell assay

HEK293–tau RD P301S FRET biosensor (ATCC CRL-3275) cells (~20,000 cells per well) were plated in 96-well black plates (Greiner Bio-One). After overnight culture, the cells were transduced for 48 hours with 3 μg/mL tau proteins (or human brain extract [P2 fraction of AD2 sample; Supplemental Figure 21]) and Lipofectamine 2000 (22, 52). FRET intensities were measured using a CLARIOstar. The converted data were normalized and calculated as described previously (53). The analysis was performed using GraphPad Prism 5 (GraphPad Software Inc.).

### Tau seeding assay in mouse primary cortical neurons

Primary cortical neuron (DIV10) cultures were performed as previously described (54). For antibody-mediated neutralization assays, neurons were pretreated with 4.5 μg/mL antibodies (Y01 or IgG) for 30 minutes. The neurons were treated with 3 μg/mL tau protein for 24 hours. Cell pellets were washed in PBS, lysed in lysis buffer, and analyzed by Western blotting.

### Cell viability assay

Primary cortical neurons (DIV10) were plated in 96-well plates and treated with 3 μg/mL tau proteins or lipopolysaccharide or transfected with tau construct according to each experiment. Cell viability was assessed using the LDH or MTT kit according to the manufacturer’s protocol as previously described (55). Briefly, after 24 hours, tau-induced toxicity was assessed by measurement of LDH activity using the CytoTox 96 Non-Radioactive Cytotoxicity Assay Kit (Promega). Cytotoxicity absorbance was measured spectrophotometrically at 490 nm in a Tecan Infinite 200. In addition, the viability of tau-treated neurons was assessed using MTT reduction assay (Sigma-Aldrich). Viability was measured spectrophotometrically by monitoring of absorbance at 540 nm in a Tecan Infinite 200.

### Binding assay of Y01 antibody with tau antigen and peptides

Affinities of antibody-antigen interactions were measured using the Octet K2 system (ForteBio), which is based on the optical principle of biolayer interferometry. The Y01 antibody was immobilized on the AR2G biosensor. Briefly, the sensor surface was hydrated by water for 10 minutes and activated by incubation for 5 minutes with a working reagent consisting of 20 mM EDC (1-ethyl-3-[3-dimethylaminopropyl] carbodiimide hydrochloride) and 10 mM s-NHS (*N*-hydroxysulfosuccinimide) in water. Y01 was immobilized on the activated biosensors in 10 mM sodium acetate buffer (pH 5.0) and quenched with 1 M ethanolamine (pH 8.0) for 5 minutes. The antibody-bearing biosensors were subjected to a baseline step of 60 seconds in PBS (pH 7.4) and then submerged for 60 seconds in wells containing various concentrations of tau peptide–conjugated BSA. To measure dissociation, the biosensors were dipped in PBS (pH 7.4) for 10 minutes. All data were evaluated using a 1:1 binding model, and binding affinity (dissociation constant) was calculated by global fitting using Octet Data Analysis (version 9.0.0.10).

### Immunohistochemistry

For staining of paraffin sections, brain tissue sections were deparaffinized in xylene and rehydrated in a graded alcohol series. Slides were rinsed in a stream of distilled water for 40 minutes for antigen retrieval. For paraffin and cryosection staining, endogenous peroxidase activity was quenched with 30% H_2_O_2_ in blocking buffer (1% normal goat serum and 0.2% Triton X-100 in PBS). The sections were then incubated with primary antibody overnight at 4°C, washed several times with PBS, incubated with secondary antibody for 1 hour at room temperature, and washed several times with PBS. Antibody information is summarized in Supplemental Table 1. Finally, sections were developed by the standard avidin-biotin-peroxidase staining method (Vectastain ABC Kit) using the DAB Peroxidase (HRP) Substrate Kit (Vector Laboratories) with nickel ammonium sulfate. For fluorescence staining, the sections were incubated with fluorescence secondary antibody for 1 hour at room temperature, washed several times with PBS, and incubated with Hoechst for 5 minutes before mounting. Stained sections were analyzed using ImageJ software (NIH).

### RAB–RIPA–formic acid extraction

Fractions of native tau proteins were prepared from tau-Tg mice as previously described (56, 57). Briefly, the hippocampus was homogenized in RAB buffer (0.1 M MES [pH 6.8], 1 mM EGTA, 0.5 mM MgSO_4_, 750 mM NaCl) supplemented with phosphatase/protease inhibitors (Complete Mini, Roche Applied Science) and centrifuged at 50,000*g* for 20 minutes at 4°C. After centrifugation, supernatant and pellet were collected separately as soluble tau and RAB pellet, respectively. The RAB pellets were suspended in RIPA buffer (0.15 M NaCl, 50 mM Tris, 0.5% deoxycholic acid, 1% Triton X-100, 0.5% SDS, 25 mM EDTA [pH 8.0], protease/phosphatase inhibitor) and centrifuged to fractionate RIPA supernatants and pellets. The RIPA pellets were further solubilized with 70% formic acid and diluted with 1 M Tris (pH 11) in a 1:20 ratio to generate formic acid supernatants. Native tau proteins in RAB or the formic acid supernatants were mixed with 2× sample buffer containing 2% SDS with or without β-mercaptoethanol and were loaded onto SDS-PAGE without boiling. Tau species were detected by immunoblotting (58).

### Preparation of Y01 Fab and 12mTau-acK280 peptide 

### for crystal formation

To prepare Y01 Fab, Y01 antibody was treated with papain (Sigma-Aldrich) at a 10:1 molar ratio in digestion buffer (PBS, pH 7.4) containing 20 mM l-cysteine (Sigma-Aldrich) and 20 mM EDTA. The reaction was incubated for 24 hours at 37°C and stopped by addition of 0.3 M iodoacetamide. The Y01 Fab fragments were then purified from Fc fragments using Protein A–agarose resin (Amicogen). Flow-through fractions containing soluble Y01 Fab fragments were collected and analyzed by SDS-PAGE. Fractions containing Y01 Fab were pooled and concentrated to about 20 mg/mL. For crystallization, concentrated Y01 Fab was mixed with 12mTau-acK280 at a molar ratio of 1:1.5. Fab and peptide complex crystals were obtained in a solution containing 68% (+/–)-2-methyl-2,4-pentanediol, 0.1 M HEPES (pH 7.5), and 10 mM calcium chloride.

### Determination of the x-ray crystal structure of the 

### Y01 Fab–12mTau-acK280 complex

Crystals of the Y01 Fab–12mTau-acK280 peptide complex were flash-cooled under a cryostream at –173°C. X-ray diffraction was performed at beamline 7A of the Pohang Accelerator Laboratory (Pohang, South Korea) (59). X-ray diffraction data were processed using HKL-2000 (60). The crystal structure of the Y01–12mTau-acK280 peptide complex was determined by molecular replacement with Phaser using search models of unliganded Fab fragments (Protein Data Bank ID 4LEX) (61, 62). Iterative cycles of model building and structure refinement were performed using Coot and Phenix, respectively (63, 64). X-ray diffraction statistics are listed in Supplemental Table 3.

### Extraction of human brain samples and fractionation

Sarkosyl-insoluble tau fractions were prepared from human brain tissue as previously described (65). Brain tissues were homogenized in ice-cold RIPA buffer (5-fold excess relative to tissue weight) containing protease and phosphatase inhibitors. Samples were cleared by centrifugation at 20,000*g* for 20 minutes at 4°C. The supernatants were transferred into new tubes, and sarkosyl (*N*-lauroylsarcosine sodium salt; Sigma-Aldrich) was added to achieve a 1% (wt/vol) solution. The reactions were incubated at room temperature for 1 hour and centrifuged at 130,000*g* for 45 minutes at 4°C. After removal of supernatants, the pellets were resuspended in ice-cold PBS, solubilized by sonication on ice, and stored at –80°C before use (sarkosyl-insoluble tau fractions). The sarkosyl-insoluble tau fractions containing native tau species of high molecular weight were assessed by Western blotting.

### Tau uptake assay in primary microglia

Primary microglial culture was maintained as previously described (66) and used to monitor tau uptake. Acetylated tau–Alexa Fluor 488 aggregates were added onto primary microglia (3 μg/mL) and incubated for 3 hours at 37°C. To observe the antibody-mediated phagocytic effect, antibodies (Y01 or IgG in 4.5 μg/mL) were added to microglia before addition of tau aggregates. Then, tau uptake by phagocytosis was examined in microglia treated with or without antibody. Cells were collected and fixed with 1% paraformaldehyde (PFA) and then resuspended in flow cytometry buffer (HBSS buffer with 2% FBS). Uptake of fluorescent tau in cells with or without antibody was quantified on a FACSCanto II (BD Biosciences) with 488 nm filters.

### Active immunization

At 3 months of age, WT and tau-P301L mice were immunized with either peptide or Adju-Phos adjuvant (InvivoGen) only. Mice were injected i.p. with 50 μg peptide mixed 1:1 (vol/vol) with Adju-Phos adjuvant (25 μg per mouse). After the first immunization, treatment was administered at intervals of 2 weeks. Injections were performed once a month. After the last injection, mice were subjected to behavioral tests. All mice were sacrificed at 8 months.

### Passive immunization

#### Intracerebroventricular infusion.

At 8 months of age, WT and tau-P301L mice were given i.c.v. injections of mouse IgG (mIgG) control or anti–tau-acK280 mIgG, mY01 (1.9 mg/mL). The pump implantation surgery was performed according to the manufacturer’s instructions. Briefly, before the surgery, an L-shaped infusion cannula was attached to catheter tubing (Alzet). A brain infusion kit was attached to a micro-osmotic pump (Alzet). The assembly pump was implanted using a stereotactic apparatus (Harvard Apparatus) into the right lateral ventricle at 0.58 mm posterior to bregma, 1 mm lateral to the midline, and 2 mm from the skull surface. An osmotic pump was subcutaneously implanted into the back of each mouse. Each pump was filled with mIgG control or mY01 (1.9 mg/mL). The osmotic pump delivered antibodies continuously at 0.11 μL/h for 28 days. Reservoir volume was 100 μL. Behavioral analysis was performed during the last 3 weeks of infusion. At 9 months of age, all mice were sacrificed.

#### Intraperitoneal injection.

At 7 months of age, WT and tau-P301L mice were injected i.p. with mIgG control or mY01 (50 mg/kg). Mice were subjected to behavioral tests. At 10 months of age, all mice were sacrificed. At 14 months of age, WT and tau-P301L mice were injected i.p. with mIgG control or mY01 (50 mg/kg). The mice were subjected to behavioral testing. At 17 months of age, all mice were sacrificed. At 5 months of age, WT and tau-P301S mice were injected i.p. with 2 different doses of mY01 (5 and 50 mg/kg) or mIgG control (50 mg/kg). At 8 months of age, all mice were sacrificed. All mice received injections weekly for 3 months. Behavioral analysis was performed during the last 4 weeks of injection.

### Tau propagation assay in tau-P301S mice

Antibodies were intravenously administered once a week to 4-month-old tau-P301S mice (weight 24–30 g). The animals received the designated antibody (control IgG or mY01) weekly starting 2 weeks before stereotaxic injection and then for 12 weeks afterward. Sarkosyl-insoluble tau fractions (P2 fraction of the AD2 sample, 3 μL, 3.3 μg/μL) were injected into the left hippocampus CA1 region (anterior-posterior: −2.3 mm; medial-lateral: 1.5 mm; dorsal-ventral: −1.8 mm). After injection using a 10 μL glass syringe (0.49 mm; Hamilton) at a rate of 0.2 μL/min, the needle was left in place for an additional 5 minutes before removal to prevent backflow of the injected material. All brain tissues were perfused with ice-cold PBS, fixed in 4% PFA (pH 7.4), transferred to 30% sucrose solution at 4°C, and frozen in OCT compound (Sakura Finetek USA Inc.). Next, coronal brain slices (30 μm) were cut with a microtome (CM1860, Leica) and stained. Four stained brain sections containing the hippocampus were quantified (equivalent to anterior-posterior, −2.25 to −2.4 from bregma). AT8-positive neurons were counted by 3 testers unaware of the treatment applied and analyzed.

### Statistics

All statistical tests were performed using GraphPad Prism 5. Detailed statistical methods and the *P* values for each comparison are listed in each figure legend, with *P* less than 0.05 considered statistically significant. Comparisons of groups were made using unpaired 2-tailed *t* tests, 1-way ANOVA with Tukey’s multiple-comparison test, and Spearman’s rank correlation. All data are expressed as mean ± SEM or SD and represent individual data values.

### Study approval

All animal procedures were performed in accordance with ARRIVE (Animal Research: Reporting of In Vivo Experiments) guidelines. All in vivo experimental protocols were approved by the Asan Institute for Life Science Animal Experimentation Committee (IACUC 2017-02-071, 2019-02-161, 2020-02-098, and 2019-02-084).

## Author contributions

HLS, NYK, DHK, and SYY conceptualized the study. HLS, NYK, JP, MIK, YNJ, SJL, KC, YLS, KHL, YSM, JAS, MSK, CGP, MJ, HJ, DLN, and MH provided methodology. HLS, NYK, JP, MIK, YNJ, SJL, KC, YLS, KHL, YSM, JAS, CGP, MJ, and MH provided investigation. HLS, NYK, JP, and MH provided visualization. MH, DHK, and SYY acquired funding. MH, DHK, and SYY provided supervision. HLS, MH, and SYY wrote the original draft of the manuscript. HLS, MH, and SYY reviewed and edited the manuscript.

## Supplementary Material

Supplemental data

## Version 1 03/14/2023

In-Press Preview

## Version 2 04/17/2023

Electronic publication
