# Supplementary material for: Monoclonal antibody Y01 prevents tauopathy progression induced by lysine 280–acetylated tau in cell and mouse models
Source: J Clin Invest. 2023 Apr 17;133(8):e156537. doi: 10.1172/JCI156537 (PMC10104896; doi:10.1172/JCI156537)
Supplement: Supplemental data [file jci-133-156537-s168.pdf]

SUPPLEMENTAL MATERIAL

Supplemental Methods

Recombinant tau proteins and peptide

Recombinant tau proteins encoding full-length tau, K18 (a tau fragment containing four-repeating MTBRs), and K18K280A (a K18 fragment with additional K280A mutation) in expression vector pET21b were produced as soluble His-tagged proteins in E.coli BL21 (DE3). Recombinant tau protein was purified by ion-exchange chromatography on an AKTA-PRIME plus fast protein liquid chromatography (FPLC) system. Purified recombinant tau proteins were assessed by SDS-PAGE and analyzed by Coomassie brilliant blue staining or western blotting. Purified tau proteins were stored at -80°C until use. 12mer peptides of tau-acK280 (12mTau-acK280, VQIIN-[ac]K-KLDLSN) used in crystallization and fluorescent polarization assays were synthesized by Peptron (Osong, Republic of Korea) and Anigen (Hwaseong, Republic of Korea). Lyophilized peptides were dissolved at 10 mg/mL in water. The peptide immunogen Tau 226-236 (phosphorylated tau threonine 231), Tau 275-286 (acetylated tau lysine 280), Tau 303-316 (acetylated tau lysine 311), and Tau 381-391 (cleaved tau glutamic acid 391) were synthesized at Anygen (Nam-myun, KOREA). N-term was conjugated with Keyhole Limpet Hemocyanin (KLH). The peptide was purified by Shimadzu HPLC 10AVP system (Purity 91.8%) with a 5-65% linear gradient of acetonitrile in 0.05% Trifluoroacetic acid (TFA).

Monoclonal antibody purification

Hybridoma cells were grown at 37°C in CD Hybridoma Medium (Gibco, Carlsbad, CA) supplemented with 8% Glutamax (1:25 dilution), 2% Ultra Low IgG FBS, and 1% penicillin/streptomycin. After 1 week, all supernatants containing antibodies were harvested from the culture by centrifugation at 13,000 rpm for 15 min at 4 °C and passed through a 0.45

μm filter (membrane filter, HM, PES04547A). Antibodies were purified using protein G Sepharose beads (Piscataway, NJ, USA) on an AKTA-start system. Antibody was eluted from the beads using elution buffer (0.1 M glycine, pH 2.7) and neutralized by supplementation with basic buffer (1 M Tris, pH 9.0). Purified antibodies were further dialyzed against PBS, pH 7.4, at 4 °C, and purity was confirmed by SDS-PAGE analysis. Purified pure antibody was stored at -20 °C until use.

DNA construct

A HA-tagged tau-WT and acetyl mutant (K174A, K274A, K280Q, K280A, K281A and K321A) were constructed by using pcDNA5 as a backbone. Point mutations and sequences of all plasmids prepared by PCR were confirmed by DNA sequencing. A pcDNA3.1-p300 were prepared from addgene (plasmid #23252).

Western blot

Samples were lysed in RIPA buffer with protease inhibitors (Sigma-Aldrich, St. Louis, MO, USA) and a phosphatase inhibitor cocktail (Sigma-Aldrich). Mouse tissues were homogenized in Pro-prep, a protein extraction solution (Intron Biotechnology, Seongnam, Korea), according to the manufacturer's instructions. The suspension was centrifuged, and the supernatant was collected. Protein concentrations were measured using the Bradford assay. For semi-denatured conditions, samples were mixed with 4× Laemmli sample buffer (62.5 mM Tris-HCl [pH 6.8], 1% [w/v] lithium dodecyl sulfate [LDS], 10% [v/v] glycerol, bromophenol blue) without β-mercaptoethanol. For denatured conditions, samples were mixed with 4× sample buffer (60 mM Tris-HCl [pH 6.8], 2% [w/v] sodium dodecyl sulfate [SDS], 25% [v/v] glycerol, 14.4 mM [v/v] β-mercaptoethanol, and bromophenol blue). All samples were heat-denatured for 5 min

at 95°C. Proteins were resolved by SDS-PAGE and transferred to polyvinylidenedifluoride membranes (Bio-Rad, Hercules, CA, USA). Membranes were blocked in 5% non-fat dry milk in PBS and 0.1% Tween-20, and incubated overnight in primary antibody at 4°C. The membranes were incubated in HRP-conjugated secondary antibodies (Vector Laboratories, Burlingame, CA, USA) for 1 hour at room temperature, and protein bands were detected by ECL (Thermo Fisher Scientific, Rockford, IL, USA). Band intensities of each sample were determined by densitometry and analyzed using the ImageJ software (NIH, Bethesda, MD, USA).

Immunoprecipitation

Homogenized lysates from brain tissues or conditioned media of donor cells were pretreated with 100 μL protein G-agarose beads (GE Healthcare Life Sciences) for 1 hour at 4°C, and then incubated overnight with the indicated antibodies. Protein G-Sepharose beads were added and incubated for 1 hour at 4°C. Brain lysates of immunized mice were incubated with protein G-agarose bead at 4°C overnight. The samples were collected by centrifugation at 6,000 rpm for 1 min at 4°C and washed twice with 0.1% Triton X-100 in PBS. All samples were resolved by SDS-PAGE and analyzed by western blot.

Behavioral tests

Nest-building

Mice were isolated in home cages a day before testing. Cotton was placed in each cage for the scoring of nesting behavior. The next day, nesting behavior was scored according to the following guidelines: score 1, cotton rarely touching and >90% intact cotton; score 2, cotton partially ripped with 50-90% intact; score 3, cotton ripped with <50% intact and spread around

the cage; score 4, cotton mostly ripped with <10% intact and gathered into side of the cage; score 5, cotton almost ripped and perfect nest shape.

Y-maze

Mice were subjected to a Y-maze test to assess short-term spatial memory. The apparatus was a Y-shaped plastic maze with three arms. Mice were habituated for 5 min. One arm (Y-shaped left arm) was open and the other arm (Y-shaped right arm) was blocked during a 5-min test. After 1 h, the other arm (Y-shaped right arm) was unblocked and behavior was analyzed for 5 min. Time in novel arm is the time to stay on the opened Y-shaped right arm.

Grip strength test

To measure strength, a weights test was performed. Animals were held by the base of the tail and lowered to the apparatus comprising a coiled wire ball for gripping. Apparatus weight was less than 3g. Time taken to drop the apparatus was measured. All tests were repeated three times.

Vertical grid test

All groups were subjected to a vertical test. Mice were placed on a wire net (horizontal, 18 cm; vertical, 30 cm). The wire net was turned over, and the time taken for mice to fall was measured. All tests were repeated three times.

Rotarod

Mice were placed on an accelerating rotarod, and exercise capacity was measured. The speed was slowly increased from 4 to 40 rpm. The time (sec) and speed (rpm) at which mice fell were

measured. Mice were trained for 4 days before the test. On the fifth day, motor test was performed. All tests were repeated 3 times.

Water maze

Mice were subjected to a water maze. The water pool was divided into four quadrants, NE, North East; SE, South East; SW, South West; NW, North West quadrants. Briefly, all mice were trained to locate a visible platform for 4 days. Training was conducted three times a day and start positions were selected as NE, SE and SW quadrants. On the 5day, the platform was removed, and movement was analyzed, such as time taken to reach the target.

Endotoxin quantification assay

The endotoxin assay was used following the manufacturer's instructions (Pierce Chromogenic Endotoxin Quant Kit, Thermo Fisher Scientific, MA, USA). The concentration of endotoxin of the diluted sample (from 1:10 to 1:100 depending on the degree of contamination) was calculated in duplicate using the standard curve (1.0, 0.5, 0.25, and 0.1 EU/mL) provided by the kit. Reaction well plates were analyzed at 405 nm using a spectrophotometer (Epoch, BioTek Instruments, Inc. VT, USA). Endotoxin is measured in endotoxin units per milliliter (EU/mL). One EU equals approximately 0.1 to 0.2 ng endotoxin/mL of solution.

Treatment with inhibitors of acetyltransferase

Cells-transfected with tau-HA or non-transfected were treated with trichostatin-A (TSA; Sigma-Aldrich, T8552) or C646 (Sigma-Aldrich, SML0002) for 24 hrs. Inhibitor stocks were prepared in DMSO and were further diluted in cell culture medium. Blank treatment constituted

of pre-treatment with 0.1% DMSO for the cells (corresponding to the same final DMSO% as for 25 μM TSA and C646).

Dot blot analyses of acetyl tau K280

The human CSF were applied to nitrocellulose membranes (BioRad, 1620112, 2 μl/samples). The Y01 antibodies were a mouse monoclonal (ADEL, 1 μg/mL) and the corresponding HRP-conjugated secondary antibody was a goat anti-mouse antibody (31430, respectively; 1/3000, Invitrogen). Finally, the membranes were incubated for five min in 3 mL/blot of ECL plus reagents (Thermo Fishers). The blots were then photographed with a digital camera (iBright FL1500), according to standard procedures and quantified the results.

Measurement of AD-derived tau uptake

BV2 microglial cells were cultured in DMEM with 2 % FBS at 5 % CO_2. Cells were pretreated for 30 min with antibodies (Y01 or IgG in 1 - 2 μg/mL) and treated with sarkosyl-soluble fractions from AD brains (S2 from Supplementay Fig 24, 3 μg/mL) for 4 hrs at 37 °C. Uptake of AD-derived tau was analyzed by semi-denaturating western blots using the anti-AT8 antibody.

Tau-acK280 capture ELISA

Anti-human-Y01(tau-acK280) monoclonal antibody (ADEL.Inc) at 10 μg/mL in sodium bicarbonate buffer (pH 9.6) was coated at 37 ℃ for 2 hr in 96-well plates (Nunc MaxiSorp; Thermo Fisher Scientific). The plates were washed three times with PBS-T(1X PBS containing 0.05 % Tween-20) and blocked with 120 μL of blocking buffer (100mM Tris, 2.5 % Casein,

0.1 % Tween-20) for 2 hrs at 37 °C with 600 rpm shaking on the plate shaker. The plates were washed three times with PBS-T and then incubated for 2 hrs at 37 °C with 600 rpm shaking on a plate shaker with serially diluted CSF from 8-month-old tau P301S mouse. Captured tau-acK280 was detected by subsequent incubation for 1 hr with 1 µg/mL (1:1,000 and 1:200 respectively) of anti-tau antibody (Abcam, ab47579) and anti-tau antibody HT7 (Thermo Fisher, MN1000) at 37 °C with 600 rpm shaking on a plate shaker. The tau antibody and HT7 recognize a mid-region epitope and did not compete for binding with anti-tau acK280 antibodies in this study. The plates were washed three times with PBS-T, followed by incubation with mixed anti mouse and rabbit IgG-HRP conjugate (1:10,000) were incubated for 1hour at 37 °C with 600 rpm shaking on a plate shaker. The plates were washed final three times with PBS-T, HRP activity was measured by incubation with TMB-ELISA substrate solution (R&D solutions) for 15 min. Developed ELISAs were stopped by the addition of 2N H2SO4 and plates were scanned at 450 nm with background at 620 nm using a microplate reader (TECAN, Infinite F50).

Rat PK studies

At 6 weeks of age, Sprague Dawley (SD) Rats (weight range 180 - 200 g) were purchased from KOATECH, Inc. On dosing day (Day 0), rats received a single intravenous (IV) dose (20 mg/kg) of The Anti-human-Y01 monoclonal antibody, and blood samples were collected on day 0 (0.5, 6 hrs after dosing) and days 1, 2, 3, 4, 6, 7, 14, 21 and 28. The blood were obtained by tail vein using 1 mL syringe and immediately placed in K2EDTA tube on 4 °C. Blood were centrifuged for 1 min at 10,000 rpm at 4 °C and the samples were stored at -80 °C until analysis. The Anti-human-Y01 mAb concentration in plasma sample was analyzed by a human IgG ELISA kit (Abcam, ab100547). The assay was performed according to manufacturer's protocol. Plates

were scanned at 450 nm using a microplate reader (TECAN, Infinite F50). PK analysis was analyzed using non-compartmental analysis of the Phoenix WinNonlin software (Ver 8.3.3, Certara).

Rat BBB permeability studies

SD Rats (6 weeks aged, weight range 180 - 200 g, KOATECH, Inc.) received third intravenous (IV) dose (20 mg/kg) of The Anti-human-Y01 mAb. Antibody was administered weekly. The blood were collected by tail vein using 1 mL syringe at 0.5 hr after first dosing. At 24 hrs after third dosing, the rats were euthanized by CO_2 asphyxiation and CSF were collected via cisterna magna puncture immediately and blood were collected via cardiac puncture. The blood were placed in K2EDTA tube and centrifuged for 1 min at 10,000 rpm at 4 °C. The separated plasma was transferred to fresh Eppendorf tube. Samples were stored at – 80 °C until analysis. For the analysis, Peptide ac-K280 at 100 ng/mL in sodium bicarbonate buffer (PH 9.6) was coated at 37 °C for 2 hr in 96-well plates (Nunc MaxiSorp; Thermo Fisher Scientific). The plates were washed three times with PBS-T (1X PBS containing 0.05% Tween-20) and blocked with 120 μL of blocking buffer (100 mM Tris, 2.5% Casein, 0.1% Tween-20) for 2 hr at 37 °C. The plates were washed three times with PBS-T and then incubated for 1 hr at 37 °C with diluted CSF and plasma from rats. The plates were washed three times with PBS-T. The Anti-human-Y01 mAb in CSF was detected by incubation for 1 hr at 37 °C with anti-human IgG-HRP conjugate at 1 μg/mL. The plates were washed three times with PBS-T and then HRP activity was measured by incubation with TMB-ELISA substrate solution (R&D solutions). Developed ELISAs were stopped by the addition of 2N H2SO4 and plates were scanned at 450 nm using a microplate reader (TECAN, Infinite F50).

Supplemental Figures

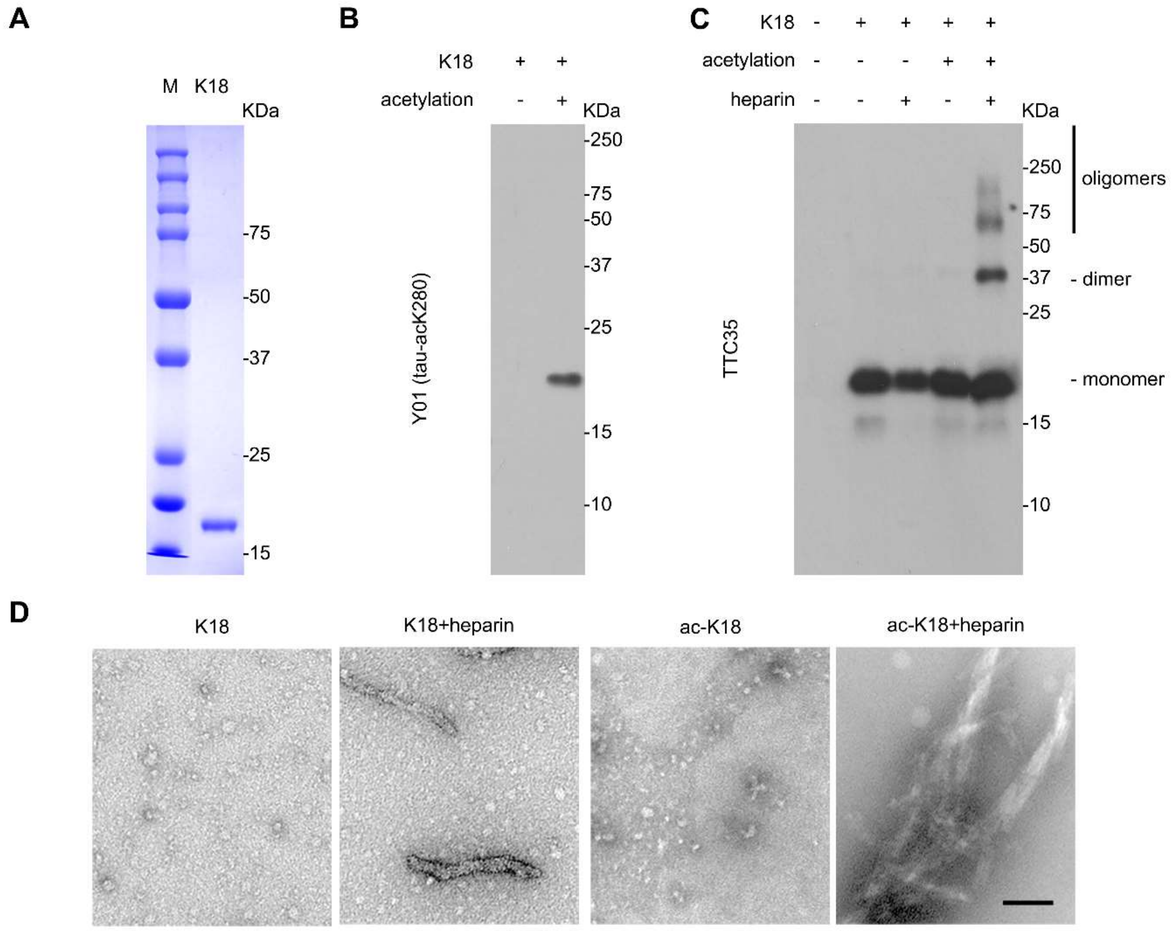

Supplemental Figure 1. Characterization of acK18. (**A**) SDS-PAGE image of K18 under reducing conditions and visualized by Coomassie stain. (**B**) Immunoblots of K18 proteins with Y01 antibody. (**C**) Immunoblots of K18 proteins with TTC35 antibody. (**D**) Electron micrograph of K18 proteins after negative staining. Scale bar: 100 nm.

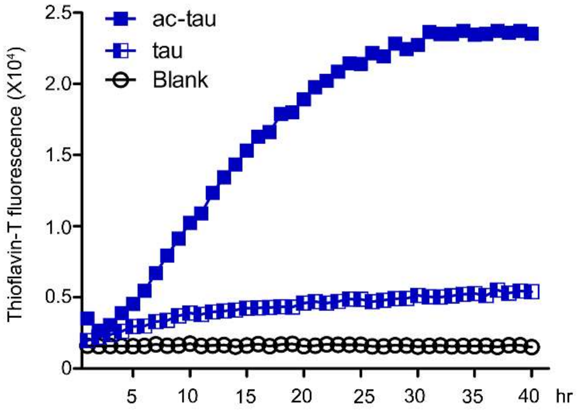

Supplemental Figure 2 Increase of full-length tau aggregation by acetylation. Tau aggregation profiles determined by thioflavin-T fluorescence using non-acetylated full-length (2N4R) tau and acetylated full-length tau (ac-tau).

A

		ng endotoxin/ml
acK18-agg	#1	<0.01
	#2	0.0689
	#3	0.0696

B **C**

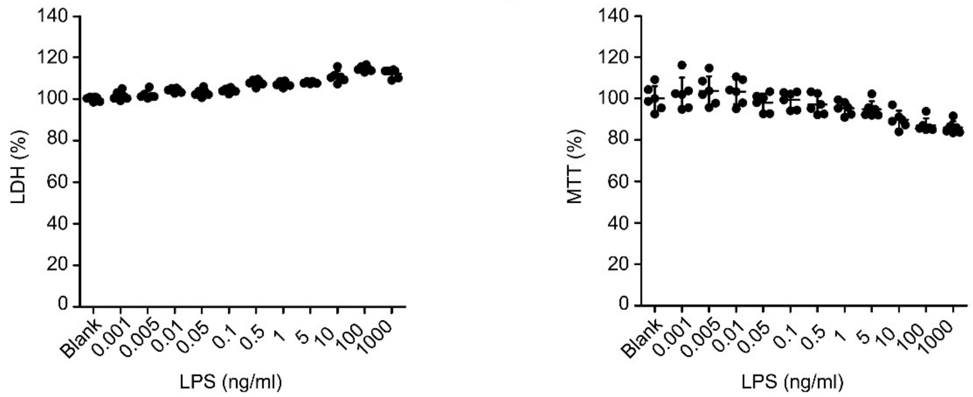

Supplemental Figure 3 Viability of neurons treated with LPS. (**A**) The endotoxin levels of final modification of acK18-agg in three independent preparations. LDH (**B**) and MTT (**C**) assays of mouse primary cortical neurons treated with LPS according to the concentrations. *n* = 6 per group. Statistical analysis was performed by one-way ANOVA followed by Tukey's multiple comparisons test. The error bars represent the SDs.

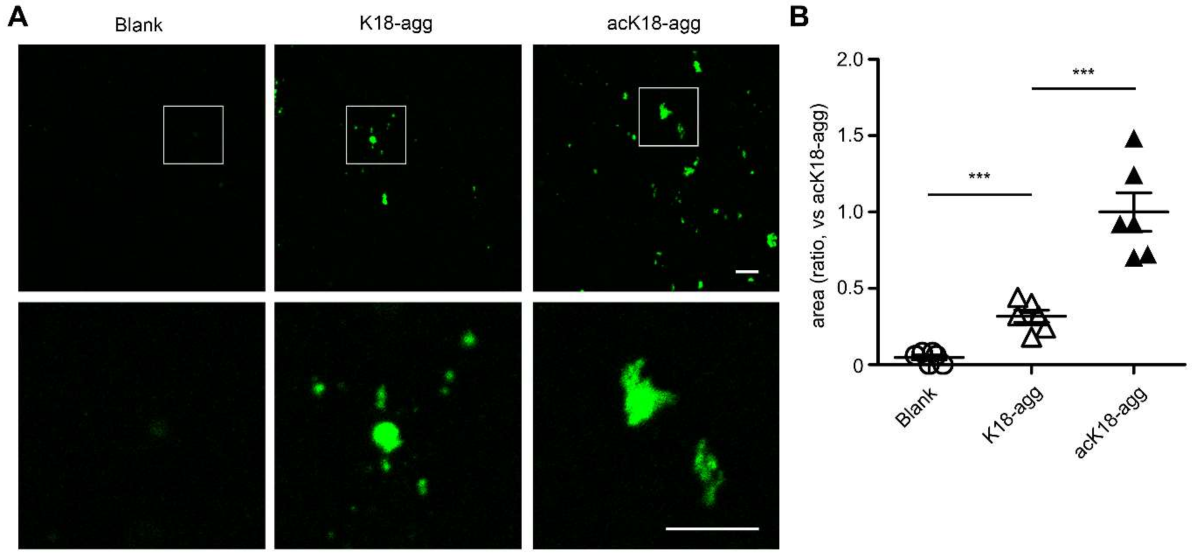

Supplemental Figure 4. Induction of tau seeding by acK18-agg in biosensor cells. (**A**) Confocal images of HEK293T tau biosensor cells treated with K18-agg or acK18-agg for 48 hrs. Scale bar = 10 μm (**B**) Quantification of fluorescence area with biosensor cells from each group. *n* = 6 per group. ***P < 0.001. The error bars represent the SEMs.

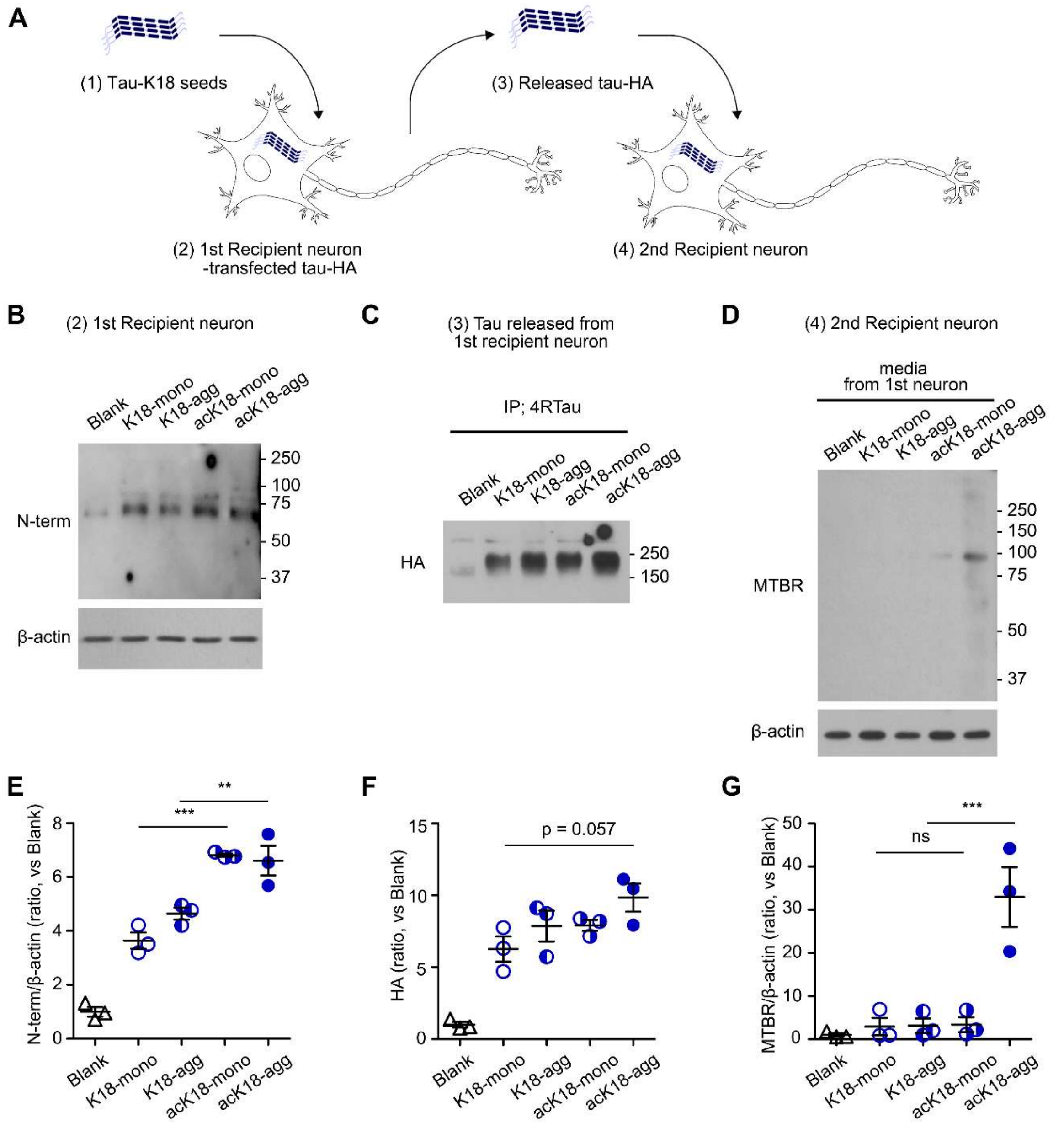

Supplemental Figure 5. Acetylation promotes the seeding and propagation properties of tau. (**A**) Schematic diagram of seeding and propagation experiment in the tau-HA-transfected mouse primary cortical neurons treated with various K18 species(1). The first recipient neurons(2) were treated for 48 hrs with equal amounts of K18-mono, K18-agg, acK18-mono, or acK18-agg, then the culture medium was completely removed, refreshed with new medium and incubated for another 24 hrs. The refreshed medium(3) containing the tau released from the first recipient neurons(2) for 24 hrs was transferred to the second recipient neurons(4) and

incubated for 48hrs. (**B**) In the first recipient neurons(2), tau was detected by semi-denatured immunoblotting using anti-Tau-N-term antibody. The upper-shifted smear bands are the most prominent in acK18-agg treated neurons. (**C**) Culture medium was immunoprecipitated with anti-4R-Tau antibody and blotted with HA antibody. (**D**) In the second recipient neurons(4), tau was detected by semi-denatured immunoblotting using anti-Tau-MTBR antibody. The upper-shifted smear bands are the most evident in the second recipient neurons treated with the medium of the first recipient neurons treated with acK18-agg. (**E**-**G**) Quantification of Tau N-term, HA and 4R-Tau levels normalized to β-actin. Statistical analysis was performed by one-way ANOVA followed by Tukey's multiple comparisons test. **$P < 0.01$, ***$P < 0.001$. The error bars represent the SEMs.

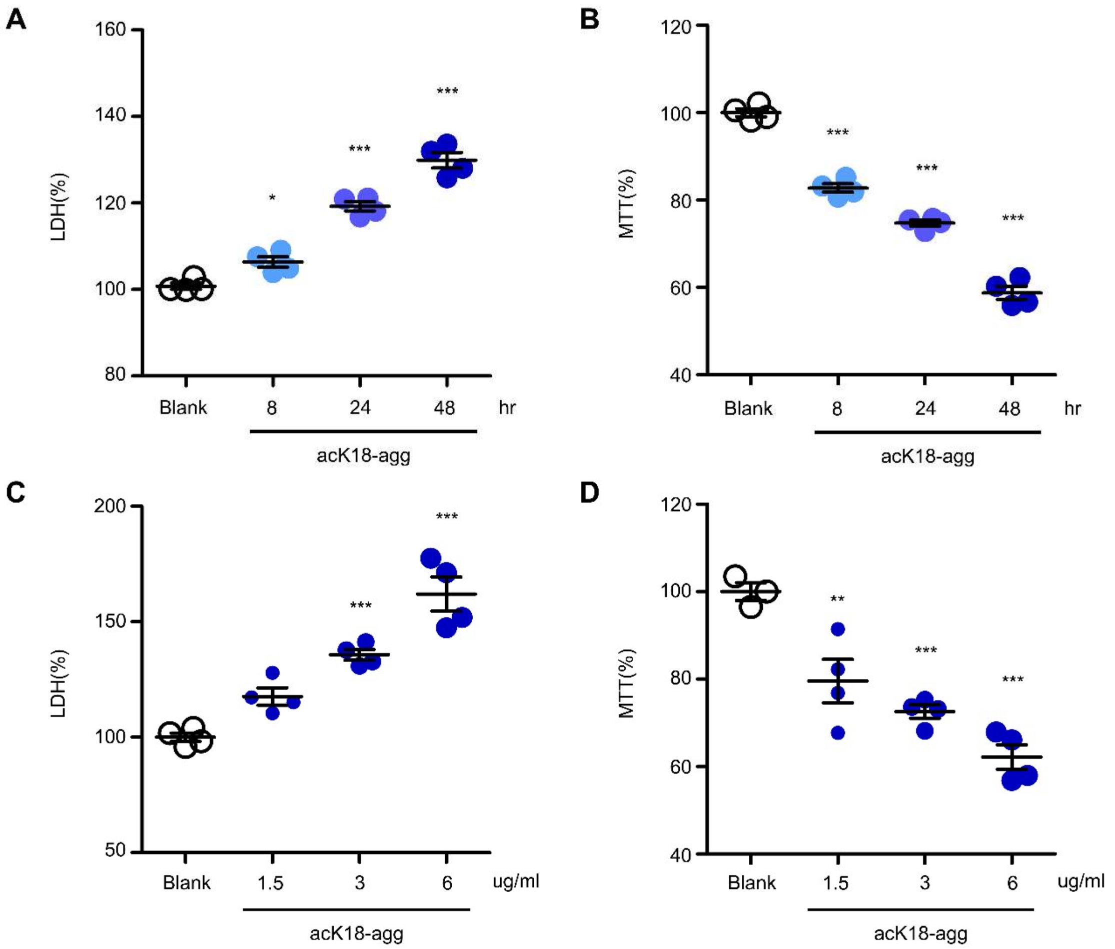

Supplemental Figure 6. Viability of neurons treated with acK18-agg. LDH and MTT assays of mouse primary cortical neurons treated with acK18-agg according to time (**A** and **B**) or concentration (**C** and **D**). *n* = 4 per group. Statistical analysis was performed by one-way ANOVA followed by Tukey's multiple comparisons test. *P < 0.05, **P < 0.01, ***P < 0.001. The error bars represent the SEMs.

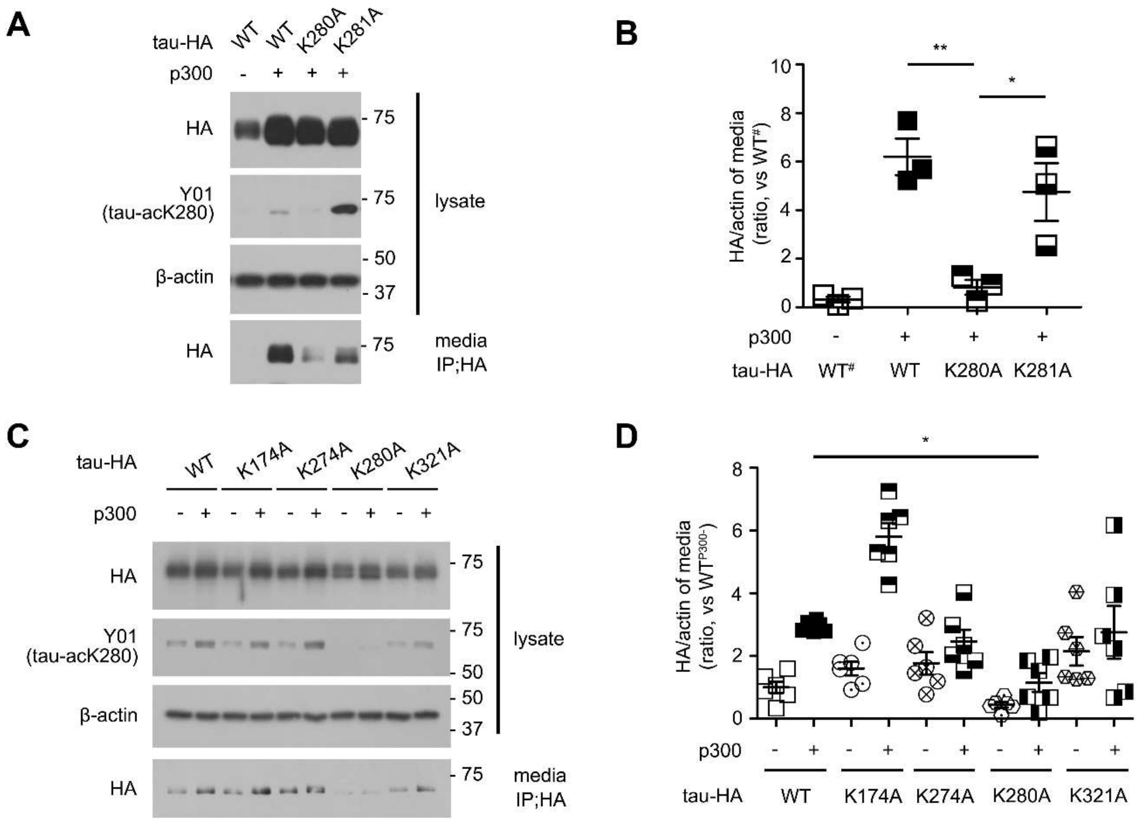

Supplemental Figure 7. Decreased release of tau by K280A mutation compared to K281A. (**A**) Representative HA and Y01 immunoblots of donor cells expressing tau-HA (WT, K280A, or K281A) acetylated by p300 acetyltransferase or tau-HA. Bottom; Representative immunoblots of HA in conditioned media; IP was performed with HA antibody. Quantification of tau-HA immunoblots of conditioned media from donor cells (**B**). *n* = 3 per group. (**C**) Representative HA and Y01 immunoblots of donor cells expressing tau-HA (WT, K174A, K274A, K280A, or K321A) acetylated by p300 acetyltransferase or not. Bottom; Representative immunoblots of HA in conditioned media; IP was performed with HA antibody. Quantification of tau-HA immunoblots of conditioned media from donor cells (**D**). *n* = 6 per group. Statistical analysis was performed by one-way ANOVA followed by Tukey's multiple comparisons test. *P < 0.05, **P < 0.01. The error bars represent the SEMs.

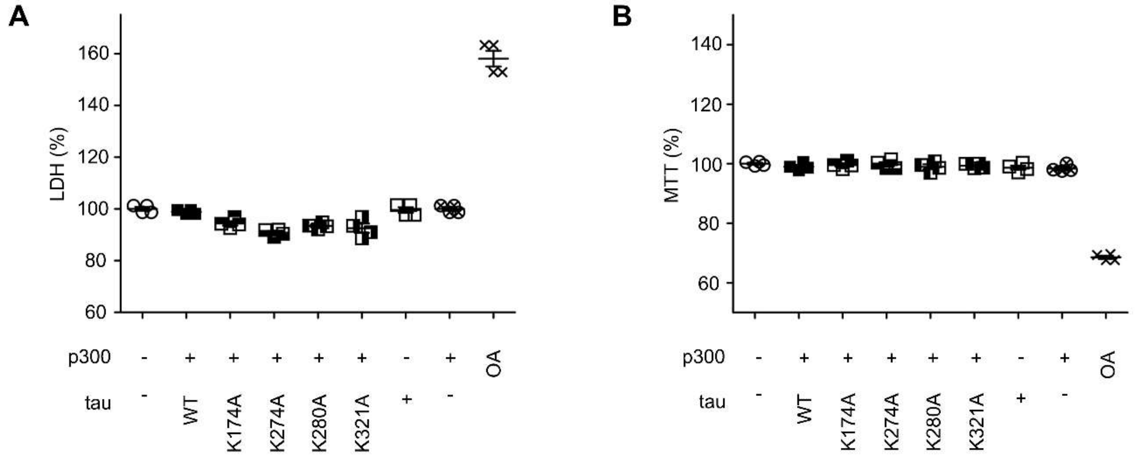

Supplemental Figure 8. Viability of transfected SH-SY5Y cells. LDH (**A**) and MTT (**B**) assays of SH-SY5Y cells transfected with tau or tau plus P300. Okadaic acid was treated to induce and compare cytotoxicity. *n* = 4 per group. Statistical analysis was performed by one-way ANOVA followed by Tukey's multiple comparisons test. The error bars represent the SEMs.

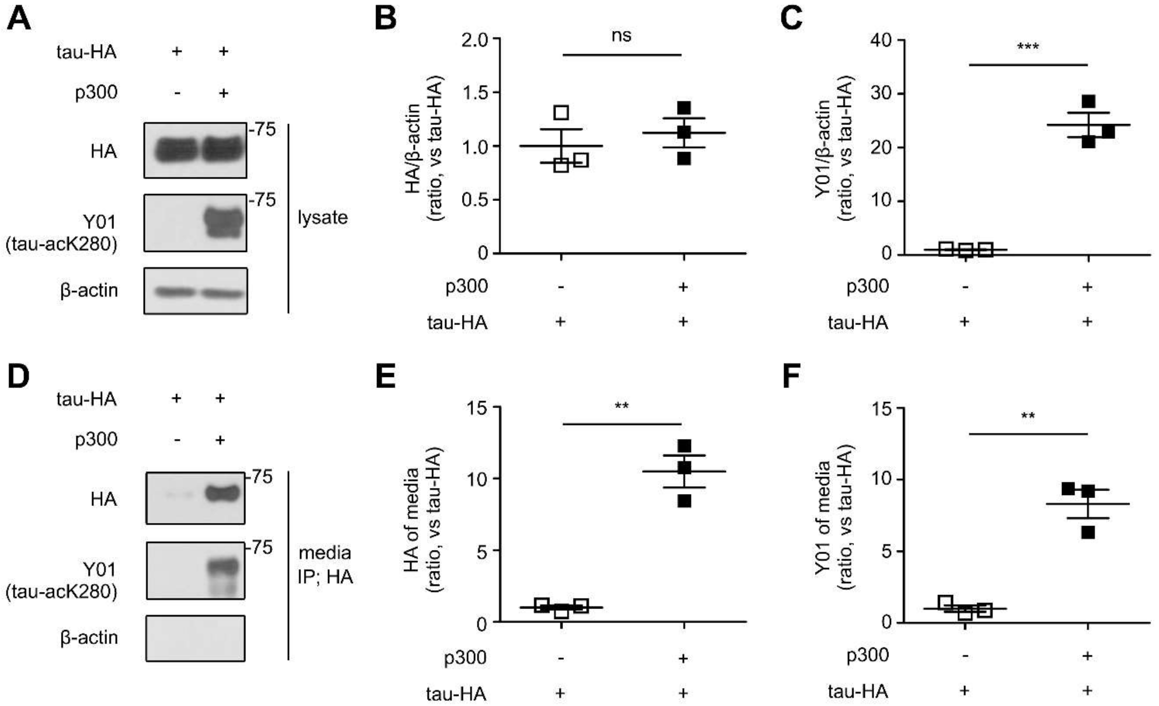

Supplemental Figure 9. Increase and release of tau-acK280 by p300 acetyltransferase. (**A**) Immunoblots of tau-HA–expressing SH-SY5Y cell lysates with or without p300 acetyltransferase. Quantification of HA (**B**) and Y01 (**C**), tau acetyl K280 levels normalized to β-actin. *n* = 3 per group. (**D**) Immunoblots of culture media from SH-SY5Y cells; immunoprecipitation (IP) was performed with HA antibody. Quantification of HA (**E**) and Y01 (**F**) levels normalized to β-actin of cell lysates. *n* = 3 per group. Statistical analysis was performed by unpaired t-test. **P < 0.01, ***P < 0.001. The error bars represent the SEMs.

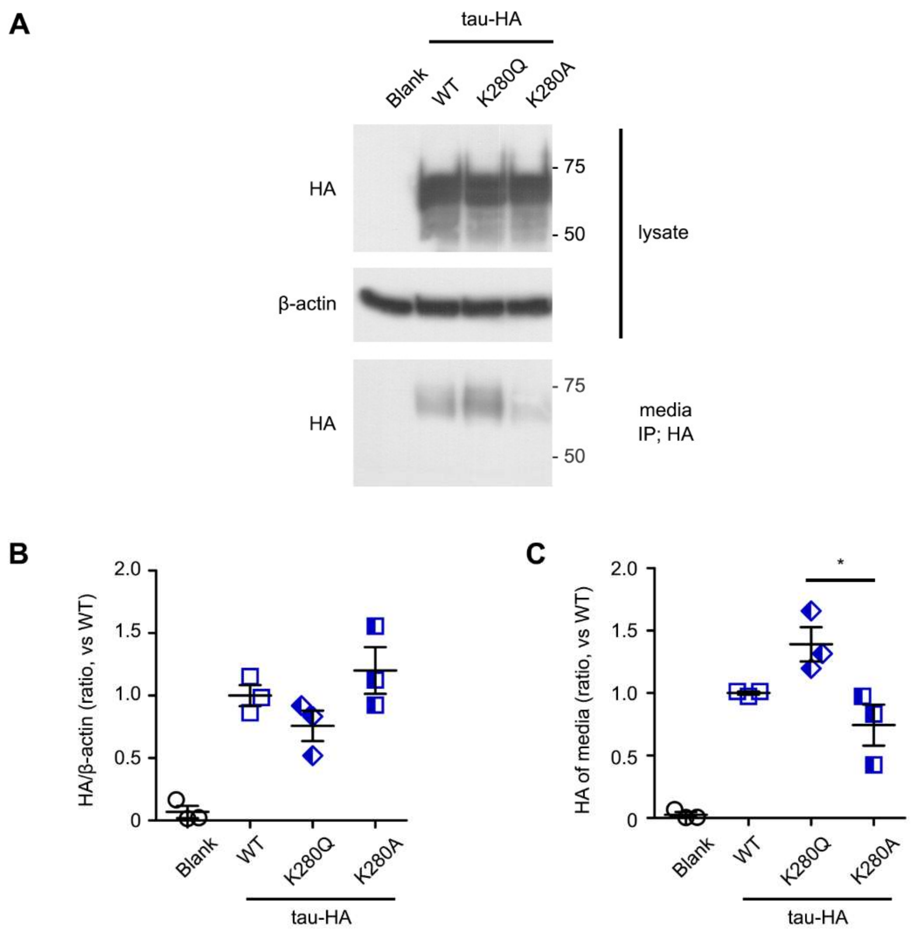

Supplemental Figure 10. Increased release of tau-K280Q. (**A**) Immunoblots of SH-SY5Y cell lysates and culture media expressing Tau-HA of wild-type, K280Q or K280A. Immunoprecipitation (IP) was performed with HA antibody. Quantification of Tau-HA levels normalized to β-actin (**B**) and HA level of conditioned media from donor cells. $n = 3$ per group. Statistical analysis was performed by one-way ANOVA followed by Tukey's multiple comparisons test. $^{**}P < 0.01$. The error bars represent the SEMs.

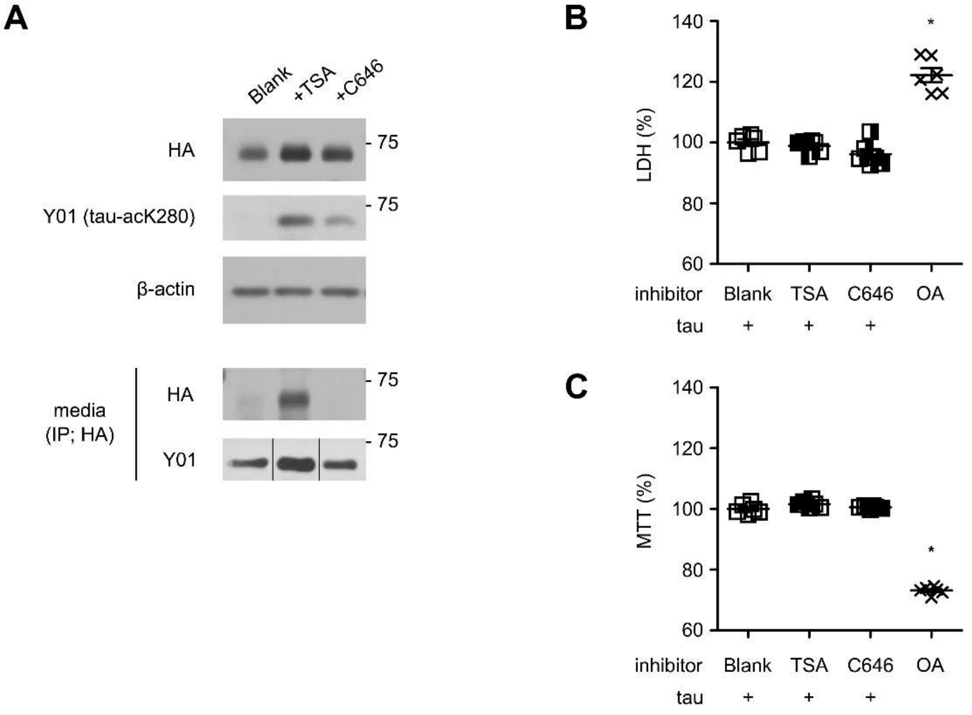

Supplemental Figure 11. No increase of tau release by inhibition of acetyltransferase. Immunoblots of mouse primary neuron lysates and culture media expressing Tau-HA (**A**). Immunoprecipitation (IP) was performed with HA antibody. The lanes of media with Y01 antibody were run on the same gel but were noncontiguous. LDH (**B**) and MTT (**C**) assays of primary neurons treated with TSA and C646, histone deacetylase inhibitor and acetyltransferase inhibitor, respectively. n = 6 per group. Statistical analysis was performed by one-way ANOVA followed by Tukey's multiple comparisons test. *P < 0.05. The error bars represent the SEMs.

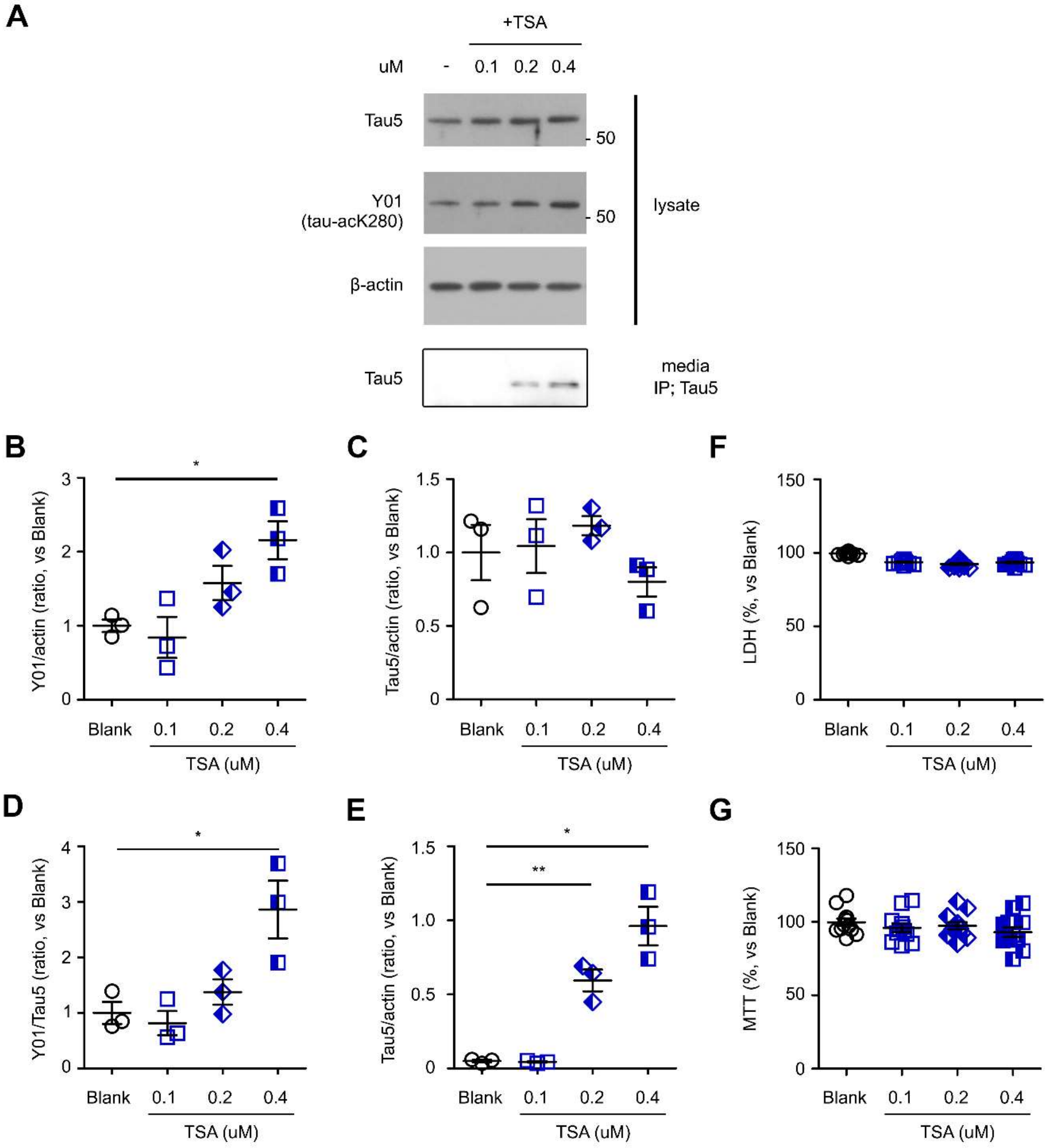

Supplemental Figure 12. Increased acetylation and secretion from primary neurons treated with trichostatin-A. Mouse primary cortical neurons were treated with histone deacetylase inhibitor, trichostatin-A (TSA) for 24 hrs with the concentrations indicated. (**A**) Immunoblots of cell lysates or culture media immunoprecipitated with tau-5 antibody. (**B**-**E**) Quantification of Tau5 and Y01 protein levels normalized to β-actin. Statistical analysis was performed by one-way ANOVA followed by Tukey's multiple comparisons test. *$P < 0.05$,

P < 0.01. The error bars represent the SEMs. (F** and **G**) LDH and MTT assays of mouse primary cortical neurons treated with TSA according to concentration. *n* = 12 per group. Statistical analysis was performed by one-way ANOVA followed by Tukey's multiple comparisons test. The error bars represent the SEMs.

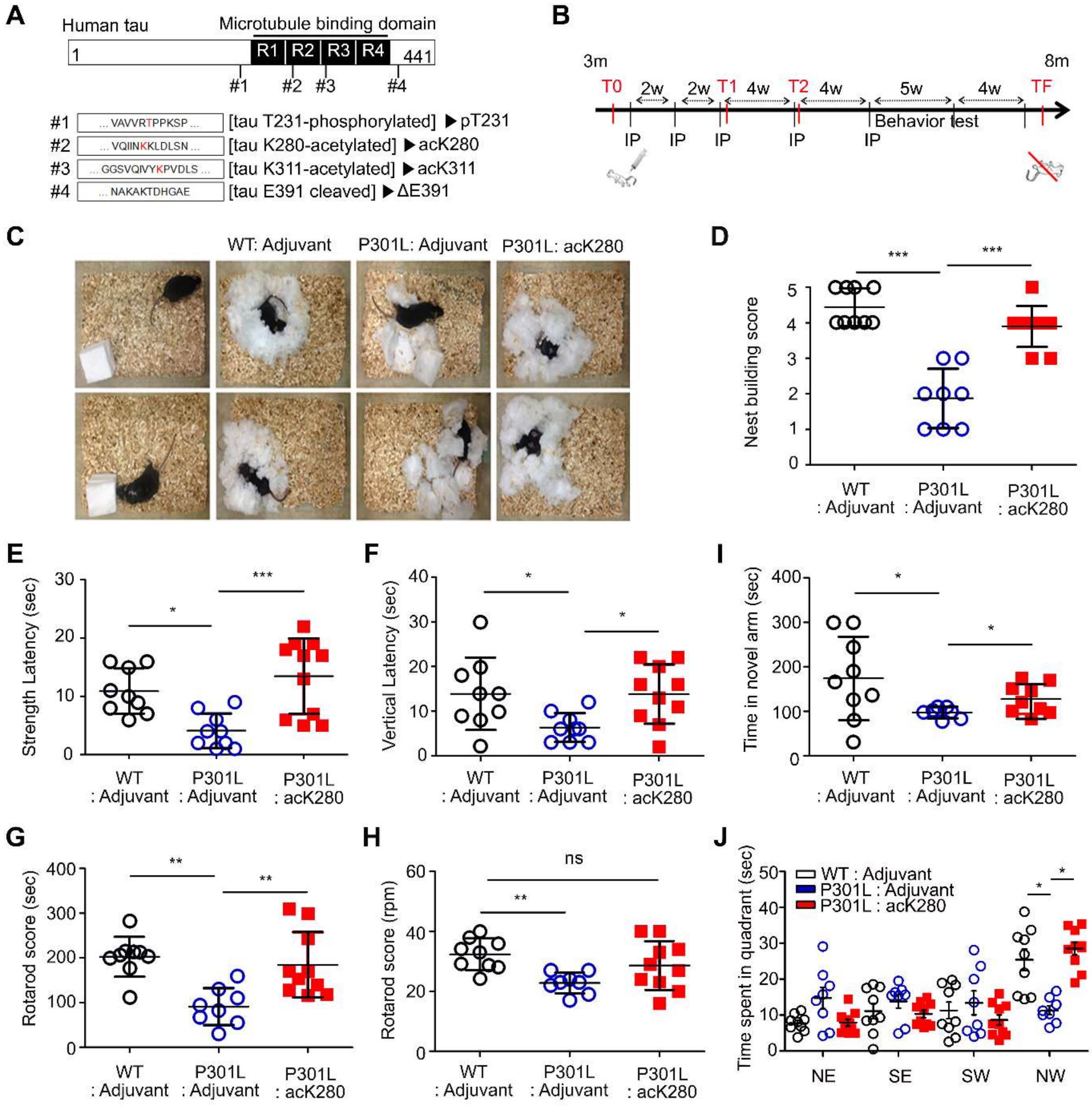

Supplemental Figure 13. Vaccination with acetylated tau at K280 ameliorates cognition in tau-Tg mice. (**A**) Epitope information used for antibody screening. #1: pT231, #2: acK280, #3: acK311, #4: ΔE391. (**B**) Schematic diagram of the active immunization experimental schedule. IP: intraperitoneal injection. T: blood sampling time. After the initial screening, behavioral efficacy with acK280 was confirmed in additional independent experiments. Representative analyzed data; acK280 peptide results (**C-D**) nest building test, (**E**) strength test, (**F**) vertical test, (**G** and **H**) rotarod, (**I**) Y-maze and (**J**) Morris water maze. Wild type +

adjuvant (n = 9), Tau P301L + adjuvant (n = 8), Tau P301L + acK280 (n = 10), all mice are male. NE: North East, SE: South East, SW: South West, NW: North West quadrants. Target quadrant: NW. Statistical analysis was performed by one-way ANOVA followed by Tukey's multiple comparisons test. *$P < 0.05$, **$P < 0.01$, ***$P < 0.001$. The error bars represent the SEMs.

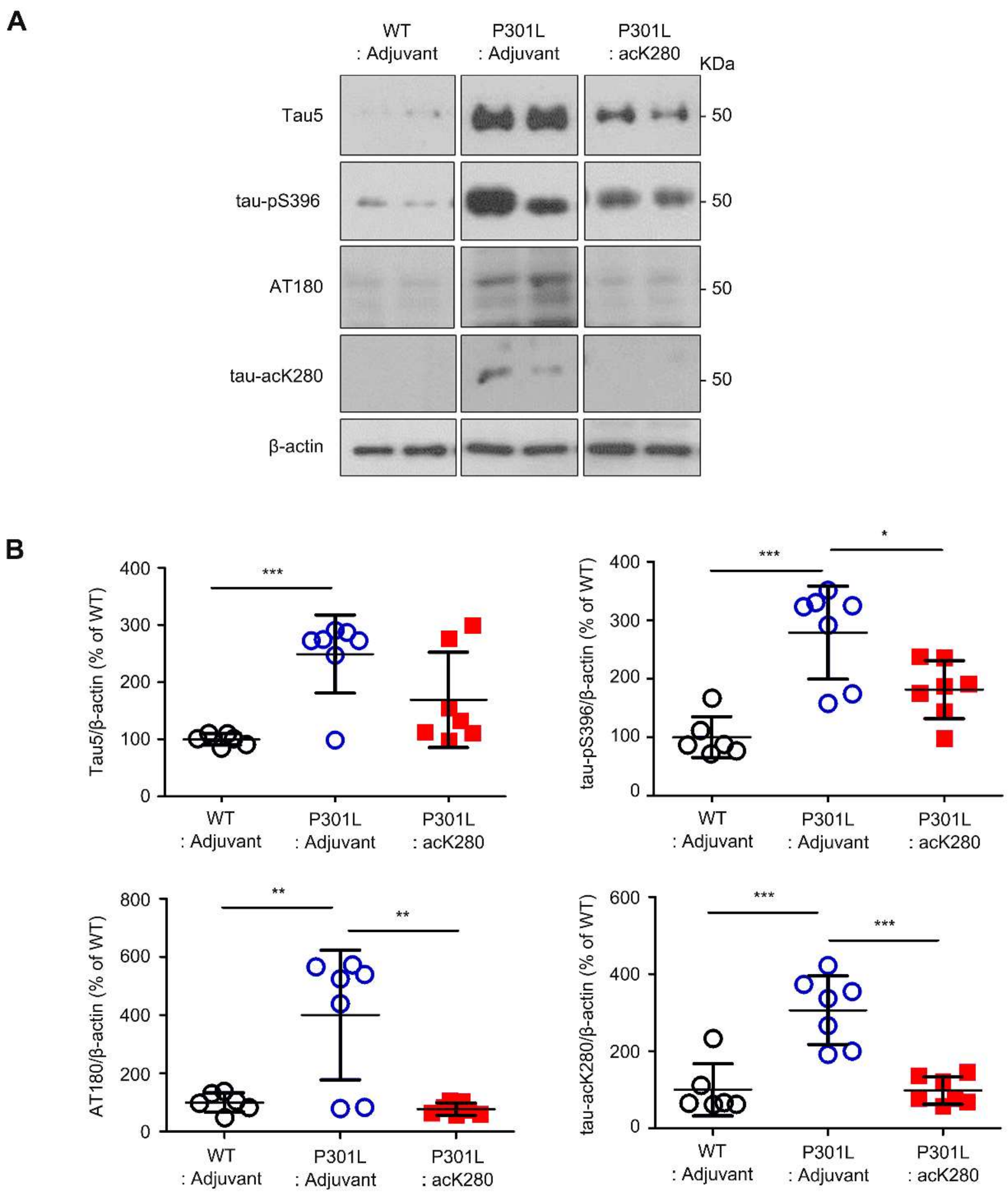

Supplemental Figure 14. Behavioral improvement by acK280 immunization Ameliorated pathology by Tau-acK280 active immunization. (**A**) Immunoblots of Tau5, pS396, AT180, Tau-acK280 (anaspec), and β-actin in the cortex from 8-month-old immunized mice. The lanes were run on the same gel but were noncontiguous. (**B**) Quantification of Tau5, pSer396, AT180, and Tau-acK280 protein levels normalized to β-actin. Wild type + adjuvant (*n* = 6), Tau P301L + adjuvant (*n* = 7), Tau P301L + acK280 (*n* = 7), all mice are male. Statistical analysis was

performed by one-way ANOVA followed by Tukey's multiple comparisons test. *$P < 0.05$, **$P < 0.01$, ***$P < 0.001$. The error bars represent the SEMs.

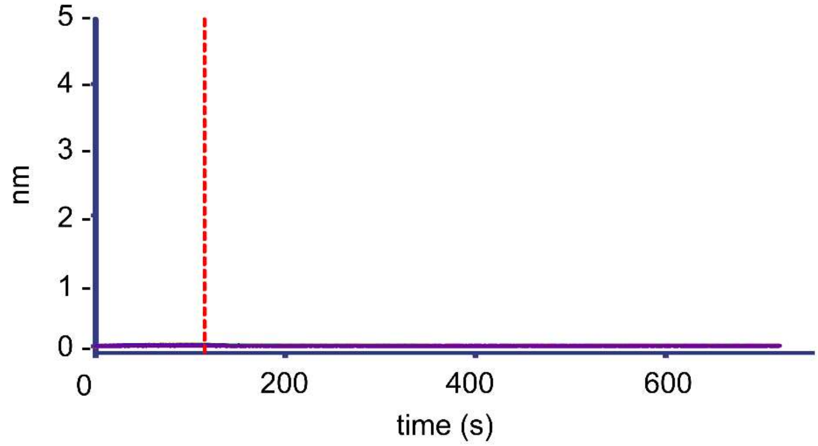

KD (M)	
Kon (1/Ms)	
Koff (1/s)	

Supplemental Figure 15. No binding affinity of Y01 for non-acetylated tau peptide. Dissociation constant between Y01 and ligand was measured by biolayer interferometry (BLI, Octet) using the 12mTau-K280 (non-acetyl) peptide as the ligand (K_D, dissociation constant; K_{on}, association rate; K_{off}, dissociation rate).

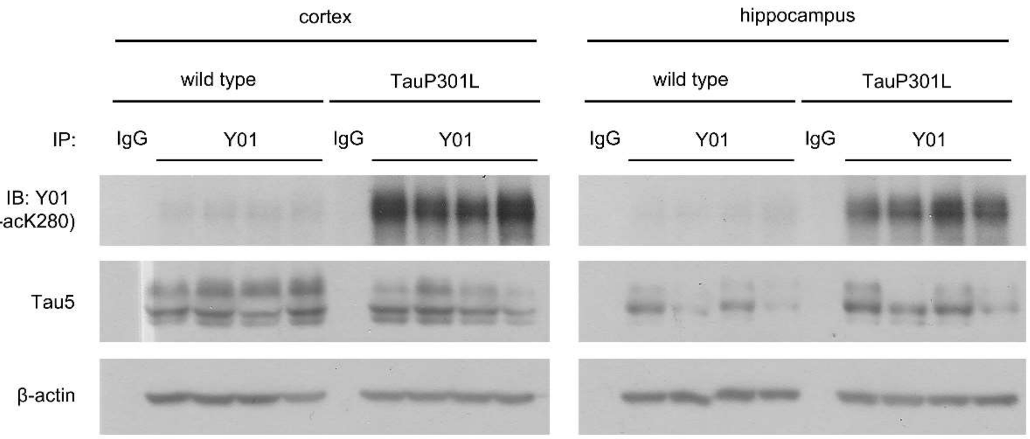

Supplemental Figure 16. Immunoprecipitation of tau-acK280 in cortex or hippocampus of WT or TauP301L mice. Immunoblots of cortex and hippocampus lysate from mouse immunoprecipitated with Y01 antibody. All mice are male.

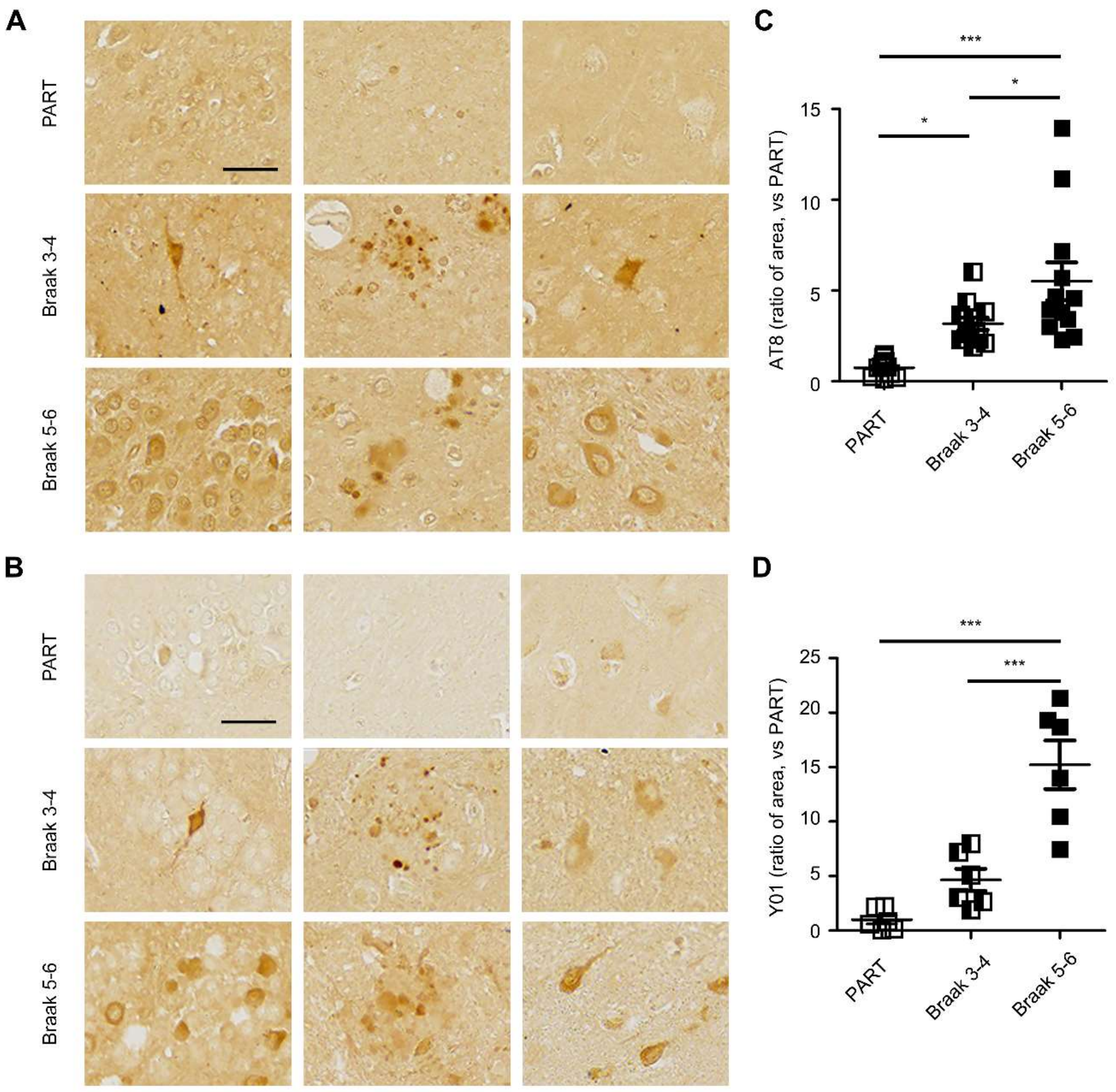

Supplemental Figure 17. Immunohistochemistry of AD hippocampus. Immunohistochemistry of AD patient hippocampus with AT8 (**A**) and Y01 (**B**) antibody. Scale bar: 100 μm. Quantification of AT8 (**C**) and Y01 (**D**) tau pathologies in AD patient hippocampus. *n* = 6 per group. Statistical analysis was performed by one-way ANOVA followed by Tukey's multiple comparisons test. *P < 0.05, ***P < 0.001. The error bars represent the SEMs.

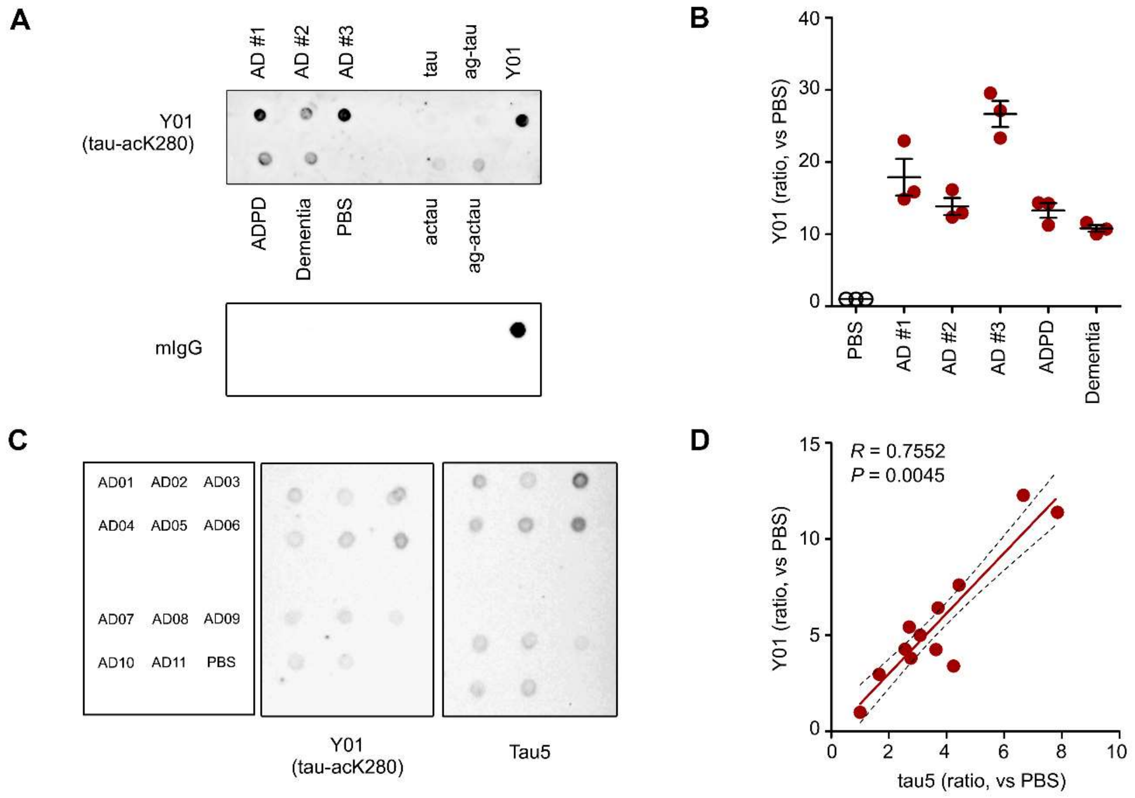

Supplemental Figure 18. Detection of tau-acK280 in CSF of AD patients. (**A**) Dot-blots of Y01 with human CSF of postmortem, PBS, recombinant tau proteins or Y01 antibody. Specific binding confirmed with dot-blots of mouse-IgG. (**B**) Quantification of Tau-acK280 protein levels normalized to PBS. Data shown are from three independent experiments. The error bars represent the SEMs. (**C**) Dot-blots of Y01 and tau5 with CSF of various patients and PBS. (**D**) Correlation between tau5 expression and Y01 levels. n = 11 (AD cases). P values were determined by Spearman's rank correlation.

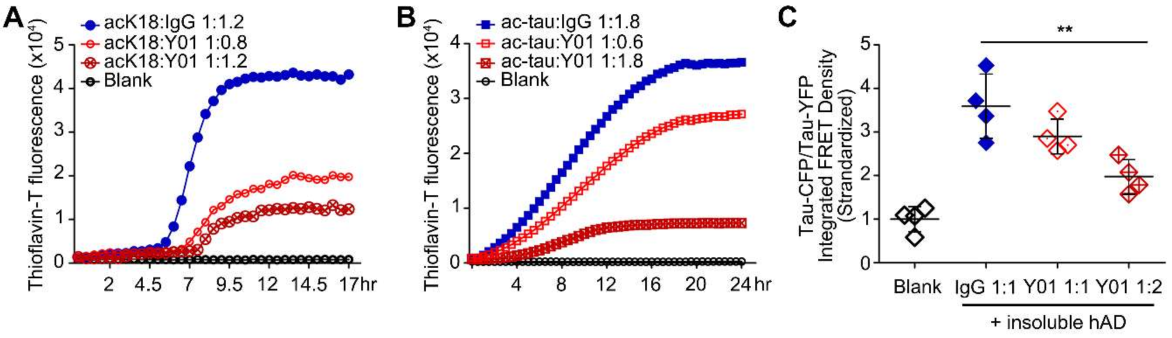

Supplemental Figure 19. Tau aggregation and seeding can be efficiently blocked by Y01. Thioflavin-T assays were performed with ac-K18 (**A**) or ac-tau (**B**) in the presence or absence of the indicated concentrations of Y01. HEK293 tau biosensor cells were incubated with the sarkosyl-insoluble fraction of human AD (**C**) in the presence or absence of the indicated concentrations of Y01, and the ratio of seeding relative to vehicle control was plotted. ac-K18; acetylated K18 tau fragment; ac-tau, acetylated full-length tau protein. Statistical analysis was performed by one-way ANOVA followed by Tukey's multiple comparisons test. **$P < 0.01$. The error bars represent the SEMs.

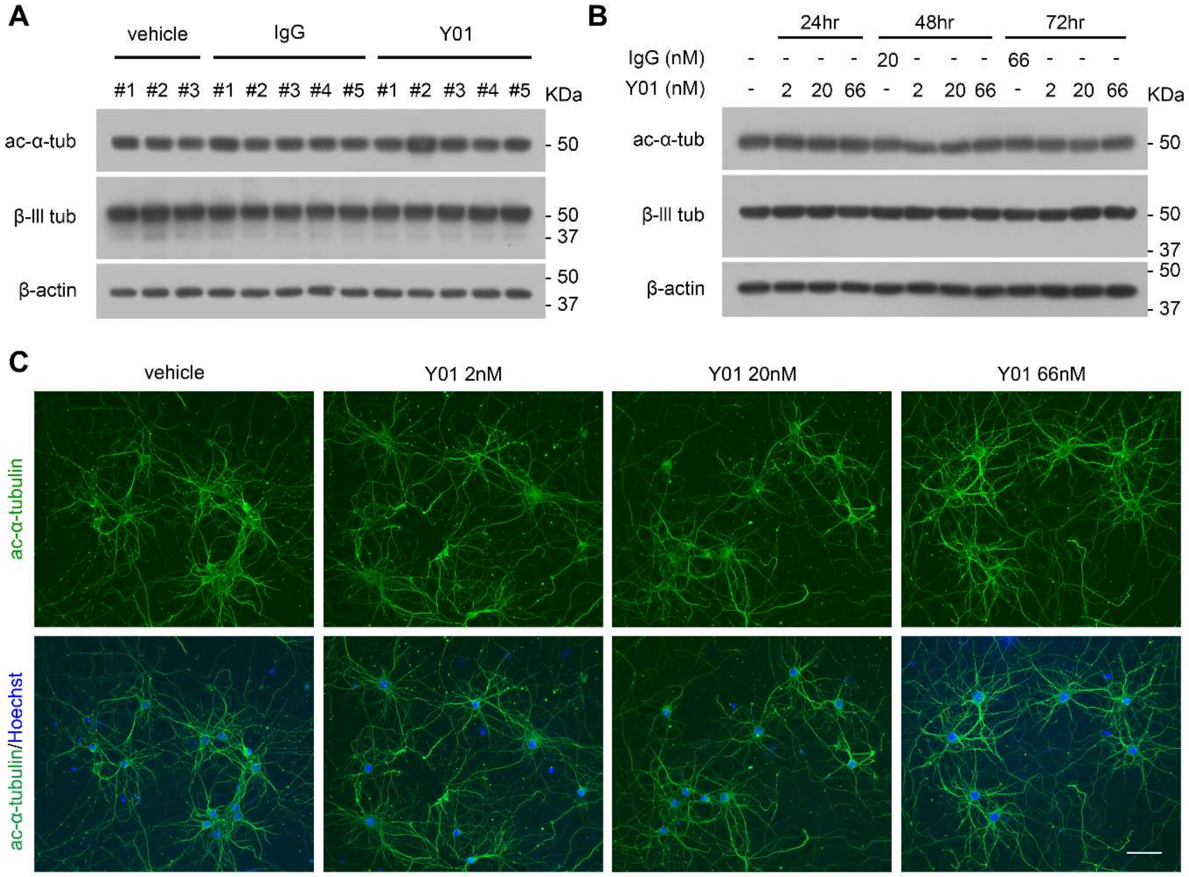

Supplemental Figure 20. Neuronal property treated with Y01 antibody. (**A**) Western blots of acetylated α-tubulin and β-III-tubulin from normal rat brains injected intravenously either with vehicle, control IgG or Y01 antibody. Vehicle (*n* = 3), IgG (*n* = 5), Y01 (*n* = 5), all rats are male. Western blots (**B**) and immunocytochemistry (**C**) of normal mouse primary cortical neurons treated with Y01 antibody.

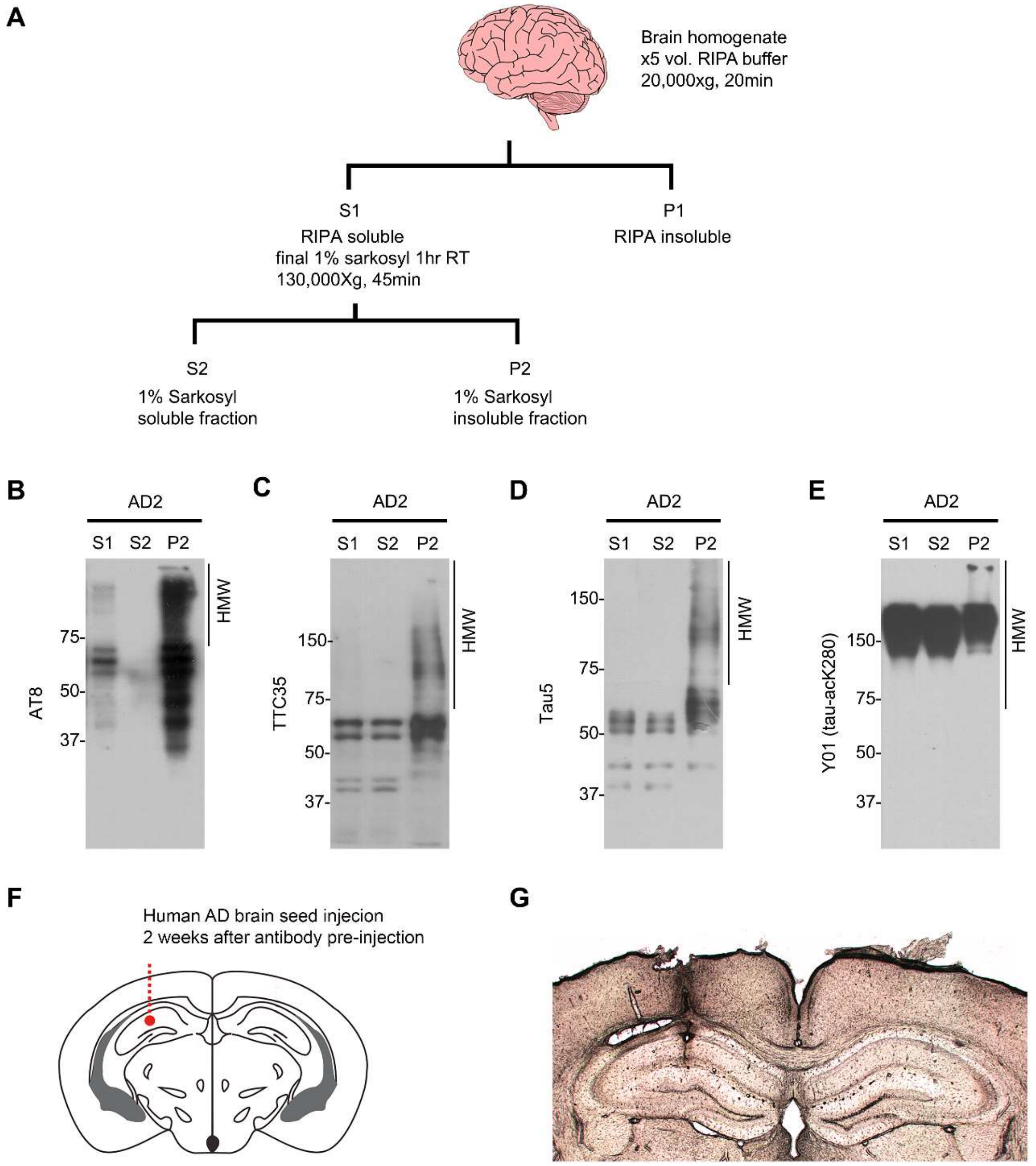

Supplemental Figure 21. Summary of the characterization of brain extract from Alzheimer's disease patients. (**A**) Workflow for preparation of the sarkosyl-insoluble fraction from human AD brain extract. AD brain extract was processed for western blotting of the sarkosyl-soluble and insoluble fractions using AT8 (**B**), TTC35 (**C**), Tau5 (**D**), and Y01-human (**E**) antibodies. (**F**) Schematic diagram of stereotaxic injections of mouse hall brain. Red dots

indicate injected sarkosyl-insoluble tau seeds. (**G**) Images showing human tau fraction-injected site. S1 = RIPA-soluble, S2 = sarkosyl-soluble, P2 = sarkosyl-insoluble. High-molecular weight tau species are indicated on the blot.

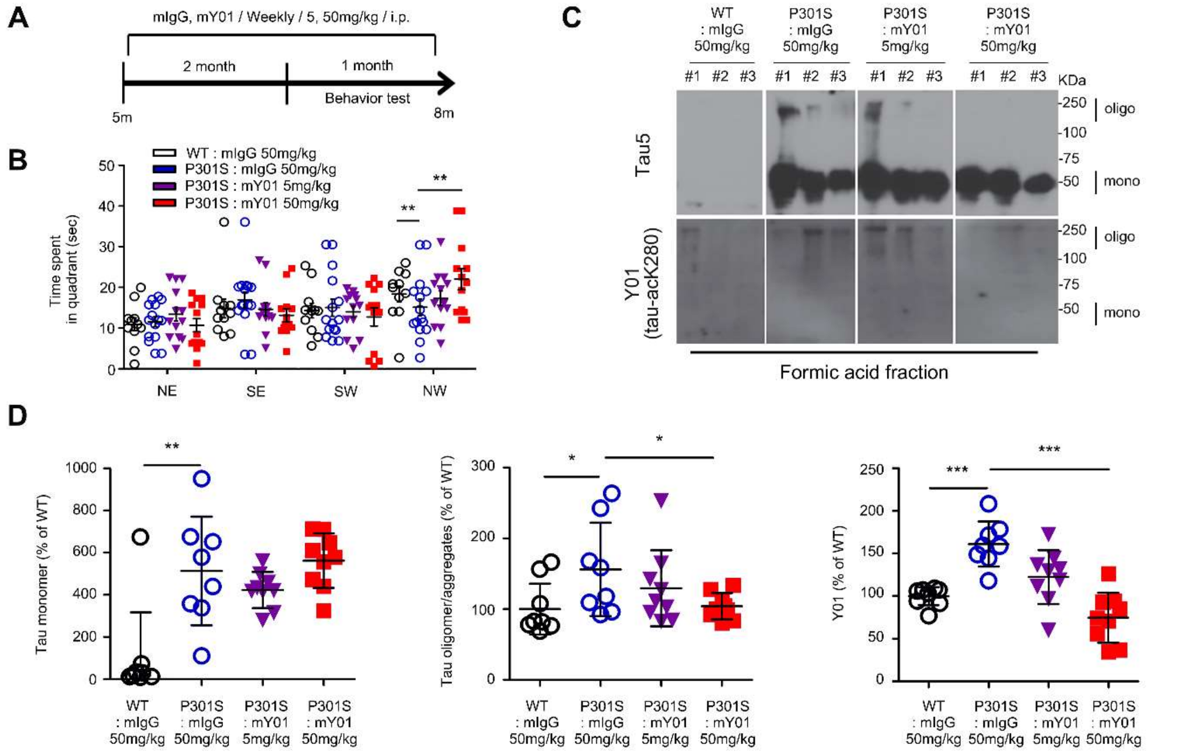

Supplemental Figure 22. Ameliorated cognition and pathology by intraperitoneal injection of mY01 in Tau P301S mice. (**A**) Schematic diagram of the passive immunization experimental design. i.p.: intraperitoneal injection. After Control mIgG or mY01 weekly injections for two months, behavioral test was performed during the next one month of weekly injections. (**B**) Morris water maze. NE: North East, SE: South East, SW: South West, NW: North West quadrants. Target quadrant: NW. Wild type + IgG 50mg/kg (*n* = 11; female *n* = 4, male *n* = 7), Tau P301S + IgG 50mg/kg (*n* = 15; female *n* = 6, male *n* = 9), Tau P301S + Y01 5 mg/kg (*n* = 13; female *n* = 5, male *n* = 8), Tau P301S + Y01 50 mg/kg (*n* = 13; female *n* = 4, male *n* = 9). (**C**) Semi-denatured immunoblots of Tau5 and Y01 protein in the hippocampus from 8-month passive immunized mice. The lanes were run on the same gel but were noncontiguous. (**D**) Quantification of Tau monomer, Tau oligomer/aggregates and Y01 protein levels normalized to GAPDH. Wild type + IgG 50 mg/kg (*n* = 9; female *n* = 4, male *n* = 5), Tau P301S + IgG 50mg/kg (*n* = 8; female *n* = 3, male *n* = 5), Tau P301S + Y01 5 mg/kg (*n* =

9; female $n = 4$, male $n = 5$), Tau P301S + Y01 50 mg/kg ($n = 9$; female $n = 2$, male $n = 7$). Statistical analysis was performed by one-way ANOVA followed by Tukey's multiple comparisons test. *$P < 0.05$, **$P < 0.01$, ***$P < 0.001$. The error bars represent the SEMs.

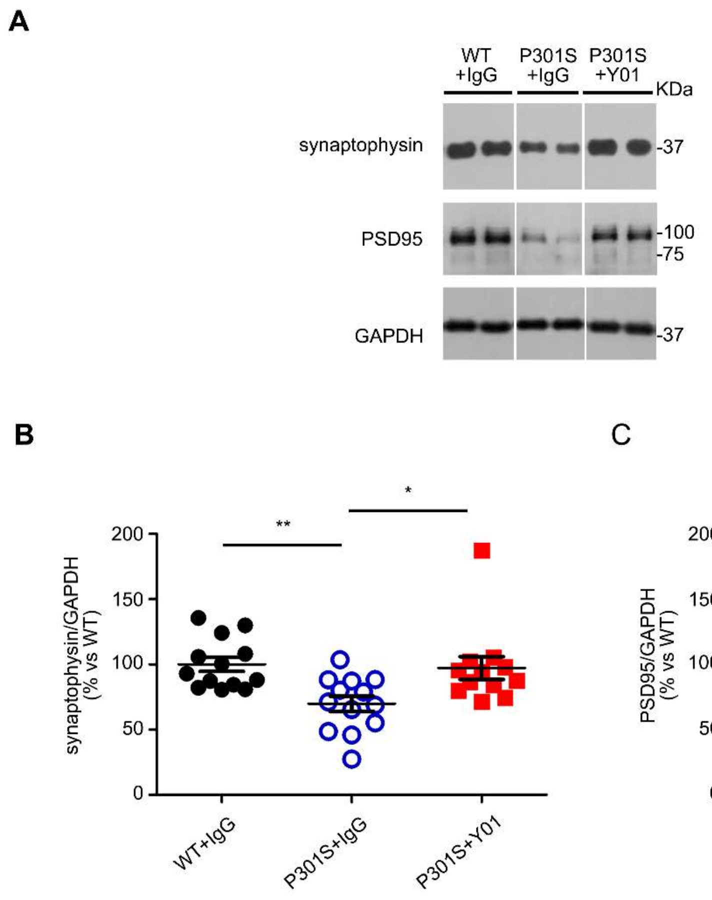

Supplemental Figure 23. Y01 enhanced the synaptic integrity in Tau P301S mice. (**A**) Immunoblots of synaptophysin and PSD95 from mouse cortex injected intraperitoneal either with 50mg/kg control IgG or Y01 antibody. The lanes were run on the same gel but were noncontiguous. Quantification of synaptophysin (**B**) and PSD95 (**C**) protein levels normalized to GAPDH. Wild type + IgG (n = 13; female n = 6, male n = 7), Tau P301S + IgG (n = 13; female n = 4, male n = 9), Tau P301S + Y01 (n = 12; female n = 4, male n = 8). Each dot represents a mouse subjected to quantitation. Statistical analysis was performed by one-way ANOVA followed by Tukey's multiple comparisons test. *P < 0.05, **P < 0.01, ***P < 0.001. The error bars represent the SEMs.

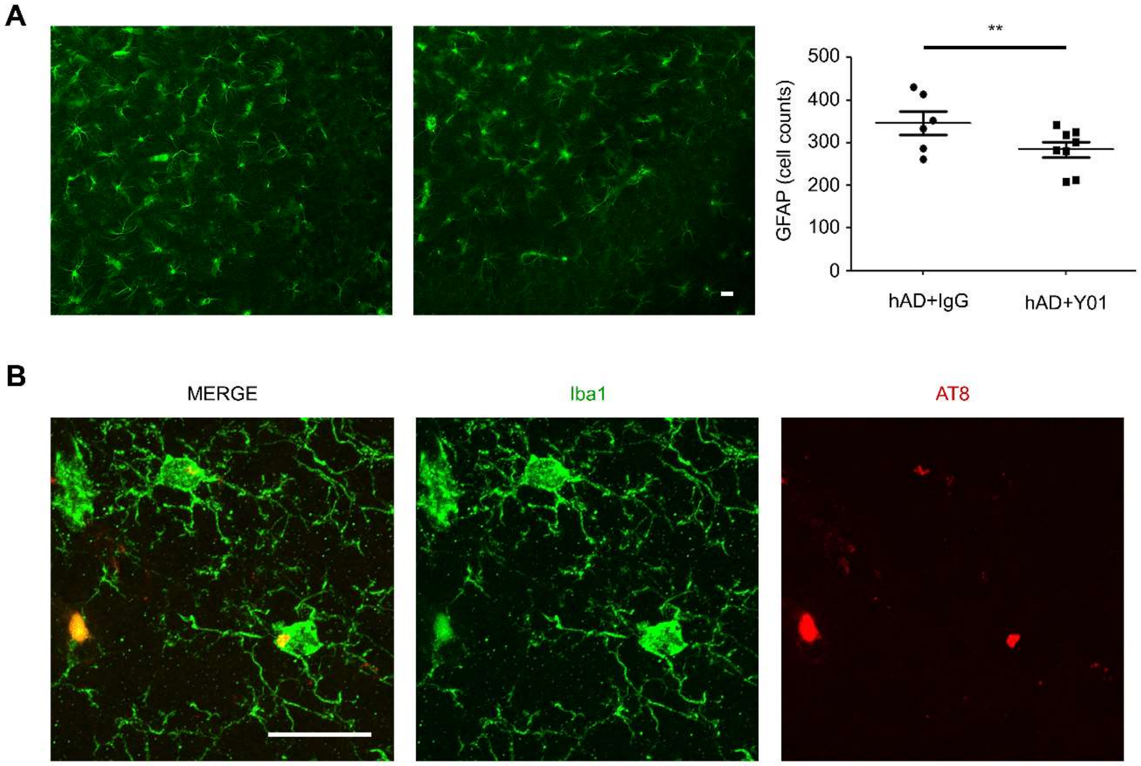

Supplemental Figure 24. Decreased GFAP-positive astrocytes in the hippocampus of Y01-injected mice. (**A**) Immunohistochemistry image of GFAP staining with Tau-P301S mouse hippocampus seeded with sarkosyl-insoluble tau fractions from AD patients. Quantification of GFAP-positive cells with coronal sections from each mice (n = 6 for IgG, n = 8 for Y01). **P < 0.01. The error bars represent the SEMs. (**B**) Immunohistochemistry image of Iba-1 (green) and AT8 (red) staining. Scale bar: 20 μm.

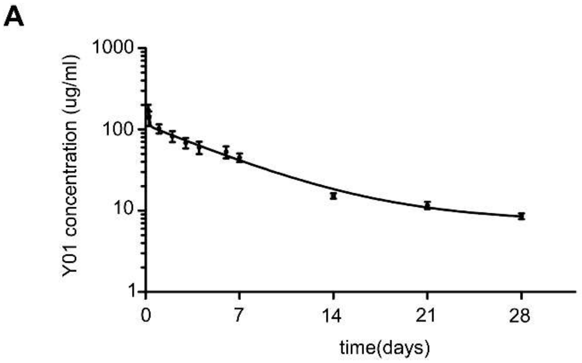

B

Name	BBB permeability (%)
#1	0.517
#2	0.173
#3	0.275
#4	0.134
Average	0.275

Supplemental Figure 25. Plasma PK profile and BBB penetration of Y01 in male SD rats. Following single intravenous administration of Y01 at 20 mg/kg to male SD rats, the maximum plasma concentration of Y01 was observed at the first time-point (0.5 hr). The mean value of clearance was 0.9 mL/hr/kg and mean value of volume of distribution at the steady state was 222 mL/kg. The mean value of Cmax was 173.8 µg/mL and mean value of AUClast was 21598 hrs*µg/mL. The mean terminal half-life (t1/2) was 185 hours. The CSF (24 hrs) to plasma (max) ratio was 0.275 %.

A

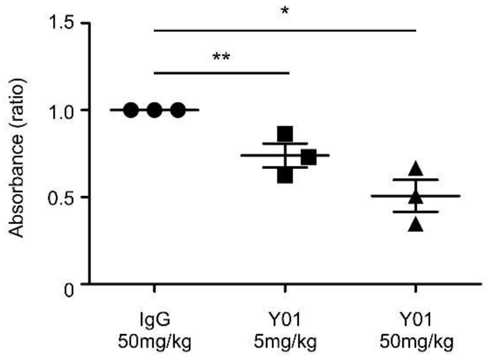

B

#1	P301S	IgG 50mg/kg	n=4
	8 month	Y01 5mg/kg	n=5
	IP	Y01 50mg/kg	n=4
#2	P301S	IgG 50mg/kg	n=4
	8 month	Y01 5mg/kg	n=4
	IP	Y01 50mg/kg	n=4
#3	P301S	IgG 50mg/kg	n=5
	8 month	Y01 5mg/kg	n=5
	IP	Y01 50mg/kg	n=5

Supplemental Figure 26. Relative levels of tau-acK280 in CSF. (**A**) Tau-acK280 ELISA was performed with the pooled CSF from 3 cohorts. Tau-acK280 level was decreased in the CSF of Y01m-injected mice. One sample t-test, *P<0.05, **P < 0.01. The error bars represent the SEMs. (**B**) Information about mice of which CSF were collected and pooled.

Supplemental Tables

Supplemental Table 1. List of antibodies.

Antibody	Features	Dilution	Source	RRID
anti-HA	polyclonal	WB(1:1000)	Sigma, H6908	AB_260070
anti-HA High Affinity	monoclonal	IP(1 µL)	Roche, 11867423001	AB_390918
anti-Tau5	monoclonal	WB(1:1000)<br>IP(1 µL)	Invitrogen, AHB0042	AB_2536235
anti-EP300/p300	polyclonal	WB(1:1000)	LSBio, LS-C372927	
Y01-mouse	acetylated tau K280 monoclonal	WB(3 µg/mL)<br>Dot blot(1ug/mL)	ADEL, Inc.	
Y01-human	acetylated tau K280 monoclonal	WB(3 µg/mL),<br>IHC(0.5 µg/mL)	ADEL, Inc.	
IgG-mouse			Bioxcell, BE0083	AB_1107784
IgG-human			Bioxcell, BE0297	AB_2687817
anti-TTC35	aggregated tau	WB(1:1000)	Rakez Kayed Lab.	
anti-acetyl lysine	acetylated lysine	WB(1:1000)	Cell signaling, 9441	AB_331805
anti-tau acK280	acetylated tau K280 polyclonal	WB(1:1000)	Anaspec, AS-56077	
anti-Tau-pS396	tau-phospho Ser396 polyclonal	WB(1:1000)	Thermo, 44-752G	AB_2533745
AT180	tau-phospho Thr231 monoclonal	WB(1:1000)	Thermo, MN1040	AB_223649
AT8 (pSer202/pThr205)	human PHF-Tau monoclonal	WB, IHC(1:2000)	Thermo, MN1020	AB_223647
N-term-human	tau N-term	WB(3 µg/mL)	ADEL, Inc.	
MTBR-human	tau MTBR	WB(3 µg/mL)	ADEL, Inc.	
4R-Tau	Monoclonal	IP(1:100)	Wako, 3E8-1A6	
anti-acetylα-tubulin	monoclonal	WB(1:1000)	Cell signaling, 5335	AB_10544694
anti-beta Ⅲ tubulin	monoclonal	WB(1:1000)	Abcam, ab18723	AB_732011
anti-GFAP	polyclonal	IHC (1:100)	Abcam, ab7260	AB_305808
anti-Iba1	polyclonal	IHC (1:100)	Wako, 011-27991	
Synaptophysin	monoclonal	WB(1:1000)	Cell signaling, 12270S	AB_2797864
PSD95 (D74D3)	monoclonal	WB(1:1000)	Cell signaling, 3409S	AB_1264242
Anti-tau	polyclonal	ELISA	Abcam, ab47579	AB_882846
Anti-tau HT7	monoclonal	ELISA	Thermo, MN1000	AB_2314654
GAPDH	monoclonal	WB(1:3000)	Invitrogen, MA5-15738	AB_10977387
Anti-β-actin		WB(1:3000)	Sigma, A5441	AB_476744

Supplemental Table 2. Information of the human samples used in this study.

**Fixed paraffin hippocampus**					
Case ID	Braak stage	Thal phase (Aβ)	Age (y)	Gender	Autopsy (h)
AD1 (NBB 2000-094)	VI	4	61	Female	196
Normal (NBB 2005-034)	0	0	56	Male	118

**Frozen tissue of frontal cortex**					
Case ID	Braak stage	Thal phase (Aβ)	Age (y)	Gender	Autopsy (h)
AD2 (KBBN03000375)	IV	3	82	male	5

**postmortem human Cerebrospinal fluid**			
Case ID	Age (y)	Gender	Autopsy
AD#1	83	Male	Alzheimer's Disease
AD#2	94	Female	Alzheimer's Disease
AD#3	69	Male	Alzheimer's Disease
ADPD	67	Female	Parkinson Disease with Dementia (PDD)<br>Alzheimer's Disease (AD)
Dementia	80	Male	Multi infarct dementia

**human Cerebrospinal fluid**			
Case ID	Age (y)	Gender	Clinical diagnosis
AD01	67	Male	Alzheimer's Disease<br>Normal Pressure Hydrocephalus (NPH)
AD02	82	Male	Alzheimer's Disease
AD03	79	Female	Alzheimer's Disease
AD04	68	Male	Alzheimer's Disease
AD05	79	Male	Alzheimer's Disease
AD06	81	Male	Alzheimer's Disease
AD07	78	Male	Alzheimer's Disease
AD08	68	Female	Alzheimer's Disease
AD09	71	Male	Alzheimer's Disease<br>Normal Pressure Hydrocephalus (NPH)
AD10	87	Male	Alzheimer's Disease
AD11	60	Female	Alzheimer's Disease

Supplemental Table 3. Crystallographic statistics of a Y01 structure complex with acetylated tau peptide.

	Y01 and acetylated tau peptide
**Data collection**	
Space group	$P2_12_12_1$
Cell parameters	a = 76.60 Å
	b = 83.86 Å
	c = 134.37 Å
Wavelength (Å)	1.00002
Resolution (Å)	50.00– 2.50
Highest resolution (Å)	2.53 - 2.50
No. observations	170,620
No. unique reflections	30.917
R_{merge} (%)[a]	7.5 (42.2)[b]
I/sigma	19.4 (5.8)[b]
Completeness (%)	99.9 (99.9)[b]
Redundancy	5.5 (5.6)[b]
**Refinement**	
Resolution (Å)	36.81– 2.50
No. of reflections (total)	30,821
No. of reflections (test)	1541
R_{cryst} (%)[c]	19.51
R_{free} (%)[d]	24.52
Average B-value ($Å^2$)	44.0
No. protein atoms	6,543
No. of ligand atoms	130
No. water molecules	6
RMSD bonds (Å)	0.011
RMSD angles (°)	1.50
Ramachandran[e] (favored)	99.0%
(outliers)	0%
PDB coordinates	7EYC.

[a]$R_{merge} = \Sigma_{hkl}\Sigma_i \mid I_i(hkl) - \langle I(hkl)\rangle \mid / \Sigma_{hkl}\Sigma_i I_i(hkl)$

[b]Numbers in parenthesis were calculated from data of the highest resolution shell.

[c]$R_{cryst} = \Sigma \mid |F_{obs}|-|F_{calc}| \mid / \Sigma|F_{obs}|$ where F_{calc} and F_{obs} are the calculated and observed structure factor amplitudes, respectively

[d]R_{free} = as for R_{cryst}, but for ~5.1 % of the total reflections chosen at random and omitted from refinement

[e]Calculated using MolProbity [23].

Supplemental Table 4. Gender information about animals used in the study.

Figure	Gender
Figure 2D	All mice are male
Figure 4, B-D	All mice are male
Figure 4, E	All mice are male
Figure 4, F and G	All mice are male
Figure 5, B-G	All mice are male
Figure 6	Female n = 4, male n = 2 for TauP301S + IgG
	Female n = 5, male n = 3 for TauP301S + Y01
Supplemental Figure 13	All mice are male
Supplemental Figure 14	All mice are male
Supplemental Figure 16	All mice are male
Supplemental Figure 20A	All rats are male
Supplemental Figure 22B	Female n = 4, male n = 7 for Wild type + IgG 50mg/kg
	Female n = 6, male n = 9 for TauP301S + IgG 50mg/kg
	Female n = 5, male n = 8 for TauP301S + Y01 5mg/kg
	Female n = 4, male n = 9 for TauP301S + Y01 50mg/kg
Supplemental Figure 22D	Female n = 4, male n = 5 for Wild type + IgG 50mg/kg
	Female n = 3, male n = 5 for TauP301S + IgG 50mg/kg
	Female n = 4, male n = 5 for TauP301S + Y01 5mg/kg
	Female n = 2, male n = 7 for TauP301S + Y01 50mg/kg
Supplemental Figure 23	Female n = 6, male n = 7 for Wild type + IgG 50mg/kg
	Female n = 4, male n = 9 for TauP301S + IgG 50mg/kg
	Female n = 4, male n = 8 for TauP301S + Y01 50mg/kg
Supplemental Figure 24	= Figure 6
Supplemental Figure 26	= Supplemental Figure 22B
